# The ability of trimethylamine N-oxide to resist pressure induced perturbations to water structure

**DOI:** 10.1038/s42004-022-00726-z

**Published:** 2022-09-28

**Authors:** Harrison Laurent, Tristan G. A. Youngs, Thomas F. Headen, Alan K. Soper, Lorna Dougan

**Affiliations:** 1 School of Physics and Astronomy, University of Leeds, Leeds, UK; 2 ISIS Facility, STFC Rutherford Appleton Laboratory, Didcot, UK; 3 Astbury Centre for Structural and Molecular Biology, University of Leeds, Leeds, UK

**Keywords:** Biophysical chemistry, Biochemistry, Biophysical chemistry

## Abstract

Trimethylamine N-oxide (TMAO) protects organisms from the damaging effects of high pressure. At the molecular level both TMAO and pressure perturb water structure but it is not understood how they act in combination. Here, we use neutron scattering coupled with computational modelling to provide atomistic insight into the structure of water under pressure at 4 kbar in the presence and absence of TMAO. The data reveal that TMAO resists pressure-induced perturbation to water structure, particularly in retaining a clear second solvation shell, enhanced hydrogen bonding between water molecules and strong TMAO – water hydrogen bonds. We calculate an ‘osmolyte protection’ ratio at which pressure and TMAO-induced energy changes effectively cancel out. Remarkably this ratio translates across scales to the organism level, matching the observed concentration dependence of TMAO in the muscle tissue of organisms as a function of depth. Osmolyte protection may therefore offer a molecular mechanism for the macroscale survival of life in extreme environments.

## Abstract (web summary)

Trimethylamine N-oxide (TMAO) protects organisms from the damaging effects of deep-sea high pressure, but it is not well understood how pressure and TMAO in combination perturb the water structure. Here, the authors use neutron scattering coupled with computational modelling of water at 25 bar and 4 kbar in the presence and absence of TMAO to propose an “osmolyte protection ratio” at which pressure and TMAO-induced energy changes effectively cancel out, which translates across scales to the organism level.

## Introduction

Extremophile organisms survive and thrive in extreme environments of salinity, temperature, pH, and pressure^1–7^. High-pressure environments on Earth are found in the deep sea, including the Mariana Trench, which reaches depths of 11 km and pressures of 1.1 kbar (8 tons per square inch)^8^. High pressure has a detrimental effect on biomolecular stability, initially causing a modest (~1%) compaction and a tendency to reduce oligomerisation of proteins at pressures of ~2 kbar^9,10^, and protein unfolding due to a shifted thermodynamic equilibrium causing more water molecules to occupy internal cavities in the hydrophobic core of the protein at ~4 kbar^11,12^ . Despite these challenges, high-pressure environments are populated by pressure-adapted organisms known as “piezophiles”^1,4,13–15^.

**Fig. 1:**
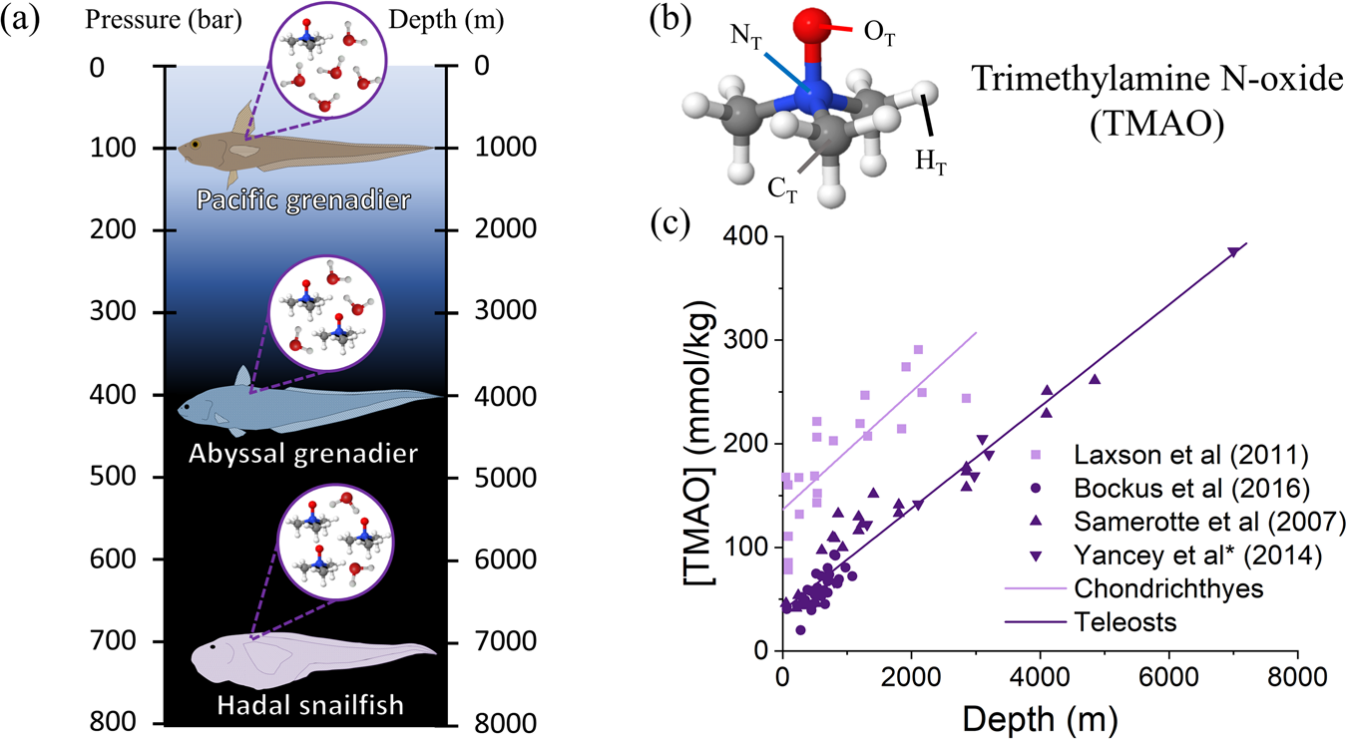
The use of TMAO as a protecting osmolyte. **a** The relationship between depth and pressure in the ocean with example teleost species that live at given depths and were used to produce the data shown in **c**. **b** The structure of the TMAO molecule, featuring a central nitrogen covalently bonded to an oxygen and three methyl groups. **c** The concentration of TMAO (measured in mmol of TMAO per kg of wet muscle tissue) in the muscle tissue of teleosts reported by Yancey et al.^26^, Bockus et al.^27^, and Samerotte et al.^28^, and chondrichthyes reported by Laxson et al.^32^ harvested from different depths. Straight line fits are added as a guide to the eye. Data for teleost species are fit by the equation $\left[{{{{{{\mathrm{TMAO}}}}}}}\right]=0.0492\times {{{{{{\mathrm{Depth}}}}}}}+39.101$. Error bars from original data are not shown for clarity.

A key adaptation strategy of piezophiles is the accumulation of organic molecules, known as “compatible solutes”, that stabilise biomolecular structures^1,16,17^. In particular, those compatible solutes that stabilise proteins against pressure denaturation are known as “piezolytes”^1,18–25^. In this work, we focus on the piezolyte trimethylamine N-oxide (TMAO). Extensive previous studies have demonstrated the important role of TMAO in high-pressure adaptation, including evidence for a measurable increase in TMAO concentration in the muscle tissue of teleosts (advanced bony ray-finned fishes)^26–31^ and chondrichthyes (jawed fish with cartilage skeletons)^32^ with increasing depth (Fig. 1).

Despite spanning different species of teleosts (cods, grenediers, eelpouts, snailfish, etc.) and different habitats (Oregon coast, Porcupine North Atlantic abyssal plain, North Pacific abyssal plain, Monterey Bay California, Hawaiian coast, and Kermadec Trench), the data are well described by a single linear fit. Such similarity in the concentration of TMAO relative to depth (and therefore pressure) across species and habitats suggests a universal protective mechanism for TMAO.

The molecular mechanism of TMAO biomolecule stabilisation is attributed to the preferential exclusion of TMAO from the biomolecule surface, resulting in preferential hydration of the molecule and maintenance of biomolecule structure^19,33–38^. Recent density functional theory calculations by Miranda-Quintana et al. have shown that protein stabilisers, including TMAO, tend to have Lewis basic properties, higher polarisability, and large dipole moments compared with protein destabilising agents^39^. Furthermore, it was shown that when the protein-solute electronegativity difference is less than the water-solute electronegativity difference, the solute is preferentially excluded from the surface, resulting in biomolecular stabilisation. In contrast, combined dynamic light scattering and molecular dynamics (MD) on aqueous TMAO and poly(N-Isopropylacrylamide) (PNIPAM), which serves as a model protein, has shown a preferential association of TMAO at the PNIPAM surface^40^. However, the TMAO hydration shell is always maintained, hence no direct TMAO-PNIPAM interaction is observed, and the solute can still be considered to be preferentially excluded. This is known as an ‘indirect’ biomolecule stabilisation mechanism as it acts upon the biomolecule through the surrounding water. Studies have explored how TMAO perturbs water structure and dynamics under ambient conditions^41–44^, under pressure^36,45–50^ and in the presence of other solutes such as urea^37,51–55^ and Mg(ClO_4_)_2_^56,57^. Combinations of MD and spectroscopy (infrared, Fourier transform infrared spectroscopy, terahertz spectroscopy, X-ray absorption) have demonstrated that TMAO forms strong hydrogen bonds with surrounding water molecules due to the large dipole moment on its NO group^34,38,43,53,55,58^. This suggests that water interacting with TMAO is more ordered than pure water^59^ and exhibits strongly retarded rotational dynamics^42,44,46^. The perturbations observed in the hydrogen bonding also extend beyond the first hydration shell of TMAO and into the bulk solvent^42,44,46^. These studies have shown reduced rotational dynamics of the bulk water due to stronger and longer-lived water–water hydrogen bonds and density fluctuations at high pressure^50^.

Despite the observed perturbations to water dynamics, the structure of water in aqueous TMAO is shown to be largely similar to pure water^36,43,45,60^. Previous studies have examined water structure in TMAO solutions, including through radial distribution functions ($g\left(r\right)$s), which describe short-range order present in liquids by relating the local density a distance $r$ from a central molecule to the global density. Classical MD^34,61,62^, ab intio MD^45,61^, and neutron diffraction^52,57^ demonstrate that the structure of water, evidenced by the water oxygen-water oxygen (O_*w*_O_*w*_) $g\left(r\right)$, is essentially unchanged in the presence of TMAO at ambient pressure.

**Fig. 2:**
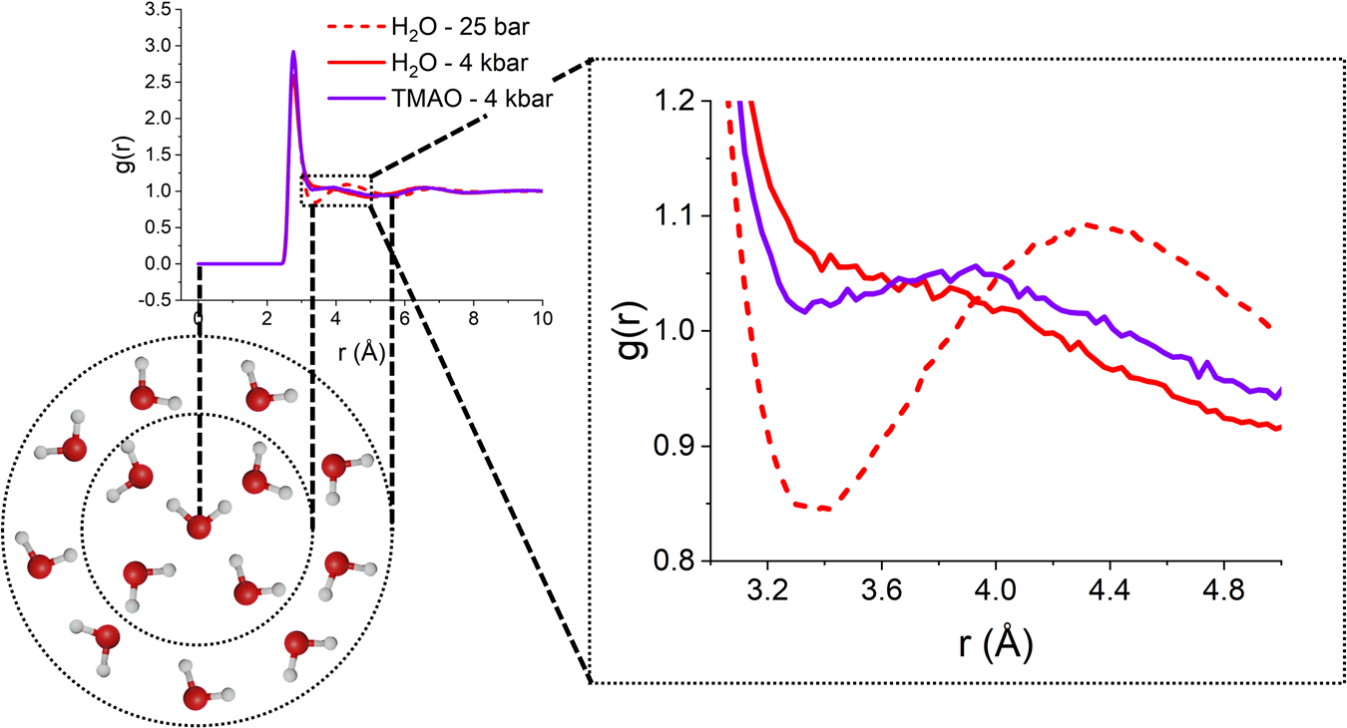
Water–water *g(r)s*. O_*w*_O_*w*_ *g*(*r*)*s* for pure water (red) and aqueous TMAO (purple) at 25 bar (dashed) and 4 kbar (solid) (Aqueous TMAO at 25 bar not displayed for clarity and can be found in the SI, Fig. S1). Schematic illustration below *g*(*r*) demonstrates how the peaks in the *g*(*r*) correspond to the hydration shells around a central water molecule.

Experimental studies on water, using both neutron^63^ and X-ray scattering^64^ and computational studies using classical^65^, first principles^66^, Monte Carlo^67^, and ab initio MD coupled with density functional theory^68^, have shown that increasing pressure results in a compaction of the second hydration shell of water into the first and weakened hydrogen bonding. This is reflected in the water oxygen–water oxygen (O_*w*_O_*w*_) $g\left(r\right)$ by the second peak moving inwards to overlap with the first peak, as can be observed in Fig. 2, while the position of the first hydration shell remains relatively insensitive. This is a result of steric effects between neighbouring water molecules and the roughly tetrahedral network structure of liquid water^69,70^. In this respect, the structure of liquid water is rather open, and water molecules in the second hydration shell around a central molecule can be compressed into the spaces left in the first hydration shell by the tetrahedrally arranged first shell neighbours^45^.

The observed opposing effects of pressure and TMAO addition on the strength of water–water hydrogen bonding prompt interesting questions about the nature of TMAO as a piezolyte. However, experimental literature on aqueous TMAO under pressure remains comparatively sparse. X-ray absorption spectroscopy studies by Knierbein et al.^50^ demonstrated that water is less compressible with increasing TMAO concentration. Fourier transform infrared spectroscopy measurements by Imoto et al.^71^ and THz spectroscopy measurements by Kolling et al.^72^ observed a blueshift of the bending/stretching modes associated with the hydrophobic methyl groups on TMAO with increasing pressure, corresponding to increased hydration. However, the modes related to hydrogen bonding to the TMAO NO group were observed to redshift, corresponding with weakened TMAO–water hydrogen bonding with increasing pressure. To gain further atomistic level structural information, these studies are supplemented with ab initio and classical MD to understand the origin of these frequency shifts^71,72^. Increased pressure results in a shift from a predominantly three-fold coordinated TMAO oxygen to an increasingly prominent 4-fold coordination. This increase in coordination results in a weakened local hydrogen bond network necessary to accommodate the extra water molecule. These results are in good agreement with modelling studies on aqueous TMAO under pressure^36,45–49^.

To gain experimentally constrained atomistic level structural information on water and aqueous TMAO under pressure, we employ neutron diffraction and the computational method of empirical potential structure refinement (EPSR). H/D isotopic substitution can be exploited to provide detailed insight into hydrogen bonding in the system^73^ and, when combined with structural refinement, can give a rich and detailed picture of particular microenvironments in aqueous solutions which are inaccessible to other ensemble averaged experimental techniques but are certainly necessary to fully understand the restructure effects of pressure and TMAO^56,57,74^.

By studying aqueous TMAO at 2.0 mol kg^−1^ H_2_O at low pressure (25 bar) and a high pressure (4 kbar), we find that water is less sensitive to pressure-induced structural perturbations when in the presence of TMAO. TMAO enhances the hydrogen bond interaction energy between water molecules at low and high pressures. We also observe that water is less compressible in the regions immediately around both the hydrophilic and hydrophobic areas of TMAO than it is outside these regions. These effects likely originate from strong hydrogen bonds formed between the TMAO oxygen and the surrounding water molecules. We observe that water–water hydrogen bonding is strengthened significantly in the hydration shell of the TMAO oxygen and moderately in the hydration shell of the methyl groups. In this respect, TMAO can be thought of as behaving like an anchor point within the water network, providing a site that can form strong hydrogen bonds from which the rest of the network can build and become more stable, as has been previously observed for other aqueous osmolytes^75^. Finally, we use the calculated values for hydrogen bond interaction energies between water molecules to determine the ratio at which the destabilising effect of external pressure is balanced by the stabilising effect of TMAO addition. We find that this ratio correlates well with experimental measurements on the concentration dependence of TMAO in the muscle tissues of organisms. These results help give atomic scale experimentally driven insight into the indirect mechanism through which TMAO acts to preserve biomolecule stability in extreme environments.

## Results

### EPSR analysis

From the EPSR simulation, refined against several isotopically distinct datasets, we observe the compression of water structure due to the large external pressure by the inward movement of the second peak in the O_*w*_O_*w*_ *g*(*r*)^63,64^. For pure water, at 25 bar the first and second peaks of the O_*w*_O_*w*_ *g*(*r*) occur at 2.77 and 4.35 Å, respectively. The first peak position is therefore consistent with water at ambient pressure, and the second peak occurs at a slightly shorter distance than the 4.5 Å peak position reported in the previous literature^76^ and likely demonstrates that even a relatively modest increase in external pressure results in a measurable structural perturbation to water.

Upon increasing pressure to 4 kbar we observe that the second peak begins to compress into the first peak, and there is no longer a discernible minimum between the two peaks. The first peak also moves slightly inwards to 2.76 Å, but as the position of this peak is strongly limited by steric effects with the central water molecule, this movement is minimal in comparison to the movement of the second peak^45,69,70^. Upon the addition of TMAO at 2.0 mol kg^−1^ H_2_O at 4 kbar, the two peak positions shift outwards compared with pure water. The first peak shows a small shift from 2.76 to 2.77 Å, and the second peak becomes resolvable again, exhibiting a peak at 3.85 Å. This indicates that TMAO is acting to preserve the structure of water against pressure-induced perturbations. The O_*w*_O_*w*_ and O_*w*_H_*w*_ *g*(*r*)s and peak positions for pure water and aqueous TMAO at 25 bar and 4 kbar are presented in Fig. S1 and Table S1 of the supplementary information.

**Fig. 3:**
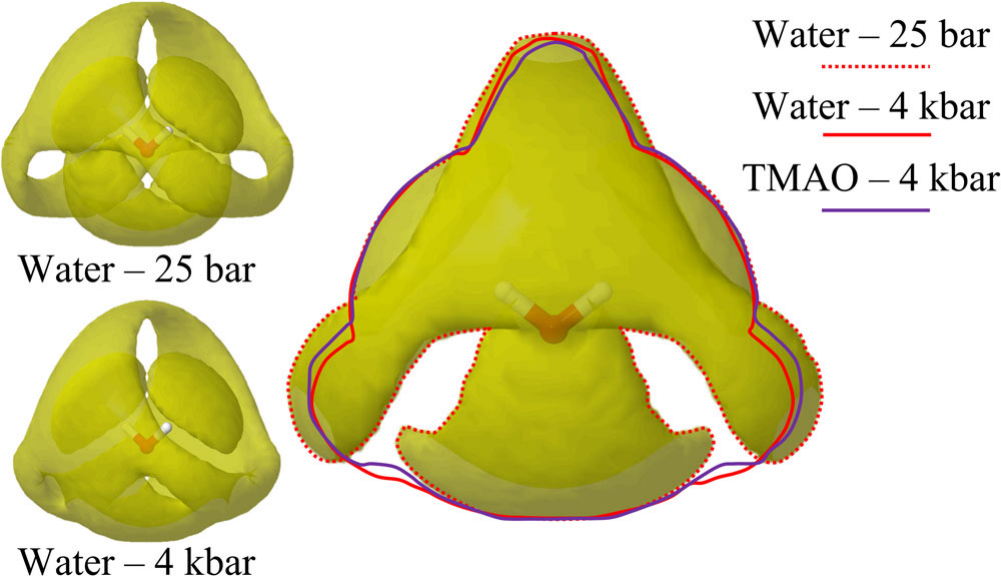
Water–water SDFs. The water–water spatial density functions (SDFs) for pure water at 25 bar and 4 kbar as viewed from 35° normal to the plane of the central water molecule. The water–water SDF for pure water at 25 bar as viewed normal to the plane of the central water molecule. This SDF is outlined (red dashed) and the outline of pure water at 4 kbar (solid red) and the outline of aqueous TMAO at 2.0 mol kg^−1^ H_2_O at 4 kbar (solid purple) are superimposed onto the image. These isosurfaces represent the 30% most probable areas of finding a neighbouring water molecule relative to a central water molecule.

The pressure-induced structural perturbation to pure water can also be visualised through the spatial density functions (SDFs) presented in Fig. 3. These SDFs can be thought of as 3D representations of radial distribution functions. The first hydration shell consists of two areas of high probability located directly above each of the two water hydrogens, and a broader area located beneath the water oxygen. The second hydration shell then fills in roughly in antiphase with the first hydration shell at a larger distance.

In Fig. 3, we again observe that the application of an external pressure causes the second hydration shell of water to be compressed into the first. This effect is most apparent in the areas of the second hydration shell directly to the left or right of the central water molecule. In this area, we notice that the outline of the SDF for pure water at 4 kbar (solid red) is closer to the central water molecule than for pure water at 25 bar (dashed red). Upon the addition of TMAO at 2.0 mol kg^−1^ H_2_O we then observe that the outline of the water–water SDF (solid purple) moves back outwards toward the water–water SDF for pure water at 25 bar. The complete SDFs for pure water and aqueous TMAO at 2.0 mol kg^−1^ H_2_O at 25 bar and 4 kbar can be found in Figs. S2 and S3 of the supplementary information. The water–water dipole angle distributions also mirror the results of the water–water SDFs and O_*w*_O_*w*_ *g*(*r*)s, as discussed in the supplementary information in notes S1 and S2, Figs. S6 and S9, and Table S6.

We can also attempt to observe the pressure-resisting ability of TMAO by examining the relative changes to the O_*w*_O_*w*_ coordination number upon increasing pressure in the presence and absence of TMAO. In this instance, the O_*w*_O_*w*_ coordination number was calculated over 3.38 Å for all four samples, corresponding to the location of the first minimum in the O_*w*_O_*w*_ *g*(*r*) at 25 bar. Here we observe that for pure water, the O_*w*_O_*w*_ coordination number increases by roughly 20% (from 4.54 to 5.44) upon increasing pressure from 25 bar to 4 kbar. However, in the presence of TMAO at 2.0 mol kg^−1^ H_2_O the O_*w*_O_*w*_ coordination number only increases by roughly 17% (from 3.97 to 4.64). Coordination numbers are listed in Table S2. This suggests that water in aqueous TMAO is less compressible than pure water and is consistent with the results of Knierbein et al.^50^. The data shows that TMAO can, to some extent, resist the pressure induced perturbation to water.

The pressure-resisting ability of TMAO is understood by considering the hydration structure of the TMAO molecule and its response to pressure. Hydration is viewed around both the hydrophilic oxygen headgroup (O_*T*_ in Fig. 1) and the hydrophobic methyl groups (centred around carbon C_*T*_ in Fig. 1). The four associated *g*(*r*)s (O_*T*_O_*w*_, O_*T*_H_*w*_, C_*T*_O_*w*_, and C_*T*_H_*w*_) are displayed in Figs. S4 and S5. These *g*(*r*)s demonstrate both areas form a structured hydration shell of neighbouring water molecules, as indicated by the presence of a clear first peak in both the O_*T*_O_*w*_ and C_*T*_O_*w*_ *g*(*r*). It is also observed that the hydration structures around TMAO are relatively insensitive to pressure. In all four of the *g*(*r*)s relevant to TMAO hydration, it is observed that the peak positions and heights remain relatively unchanged as pressure is increased from 25 bar to 4 kbar when compared with the O_*w*_O_*w*_ and O_*w*_H_*w*_ *g*(*r*)s. Again, this is reflected in the TMAO–water dipole angle distributions presented in the supplementary information in Figs. S7 and S8 and discussed in notes S1 and S2.

We can also demonstrate reduced compressibility of water around the TMAO molecule through the O_*T*_O_*w*_ and C_*T*_O_*w*_ coordination numbers, calculated over 3.38 and 4.48 Å, respectively. Upon increasing pressure from 25 bar to 4 kbar the O_*T*_O_*w*_ coordination number increases by 15% (from 2.65 to 3.04), and the C_*T*_O_*w*_ coordination number increases by 17% (from 8.10 to 9.44), broadly consistent with the previous literature^36,45,50,72^. Coordination numbers are listed in Tables S4 and S5. In both instances, this is lower than the 20% increase in the O_*w*_O_*w*_ coordination number for pure water.

**Fig. 4:**
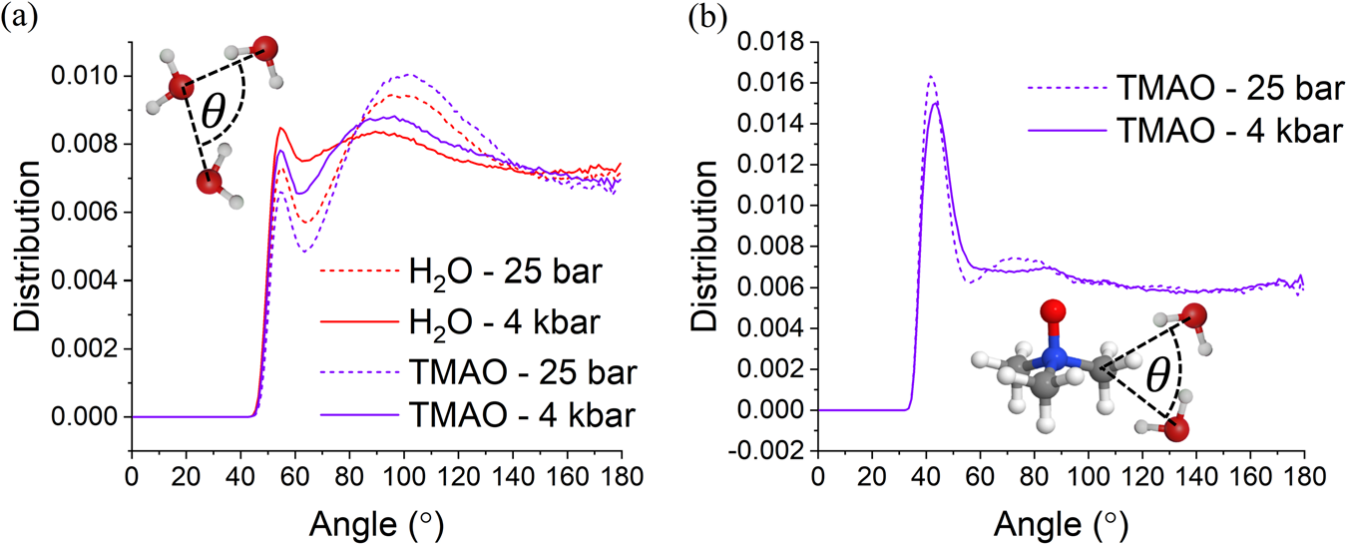
Assessing tetrahedriality of water around water and TMAO. Triplet angle distributions for O_*w*_O_*w*_O_*w*_ (**a**) and O_*w*_C_*T*_O_*w*_ (**b**) triplets. Three atoms are part of a triplet if the two O_*w*_ atoms are within a 3.38/4.48 Å from a central O_*w*_/C_*T*_ atom which forms the apex of the triangle. The calculated angles are normalised to the sin θ dependence that would occur for a random distribution of angles^73^.

The low compressibility of water around O_*T*_ compared with pure water can be explained by strong hydrogen bonds between O_*T*_ and the surrounding water molecules, which have been well observed in the previous literature^34,42–44,53,56^; however, the low compressibility of water around the methyl groups compared with pure water cannot be similarly rationalised. This is instead rationalised by considering the origin of the compressibility of pure water. As discussed in the introduction, the tetrahedral nature of the water molecule itself leads to a relatively open tetrahedral network. This can be demonstrated through the O_*w*_O_*w*_O_*w*_ triplet angle $\theta$ distribution shown in Fig. 4a. Three O_*w*_ atoms are part of a triplet if two O_*w*_ atoms are within 3.38 Å from a third central O_*w*_ atom. In these distributions, we observe two peaks. In H_2_O at 25 bar these peaks occur at 98.9° and 55.0°, representing the angle between a central O_*w*_ and two tetrahedrally ordered first shell O_*w*_ neighbours (close to the ideal tetrahedral bond angle of 109.5°) and a single first shell O_*w*_ neighbour and a single second shell O_*w*_ neighbour and a single second shell O_*w*_ neighbour, respectively. Increasing pressure to 4 kbar causes the low angle peak to increase in amplitude as more second shell neighbours are compressed into the first hydration shell, and the large angle peak moves inwards to 91.0° while decreasing in amplitude as the first hydration shell is distorted. TMAO addition causes the opposite effect as hydrogen bonding and tetrahedrality of the network are enhanced. Conversely, the O_*w*_C_*T*_O_*w*_ triplet angle distribution (calculated according to a C_*T*_O_*w*_ distance of 4.48 Å) only shows a single low angle peak located at ~43°. This suggests that water around a central TMAO methyl group does not form the same open tetrahedral structure as it would do around a central water molecule. This, in turn, means that the immediate network around the methyl group will be less compressible than the remaining water, as there will be fewer free spaces which second hydration shell molecules can occupy.

### Quantifying hydrogen bond interaction energies

**Fig. 5:**
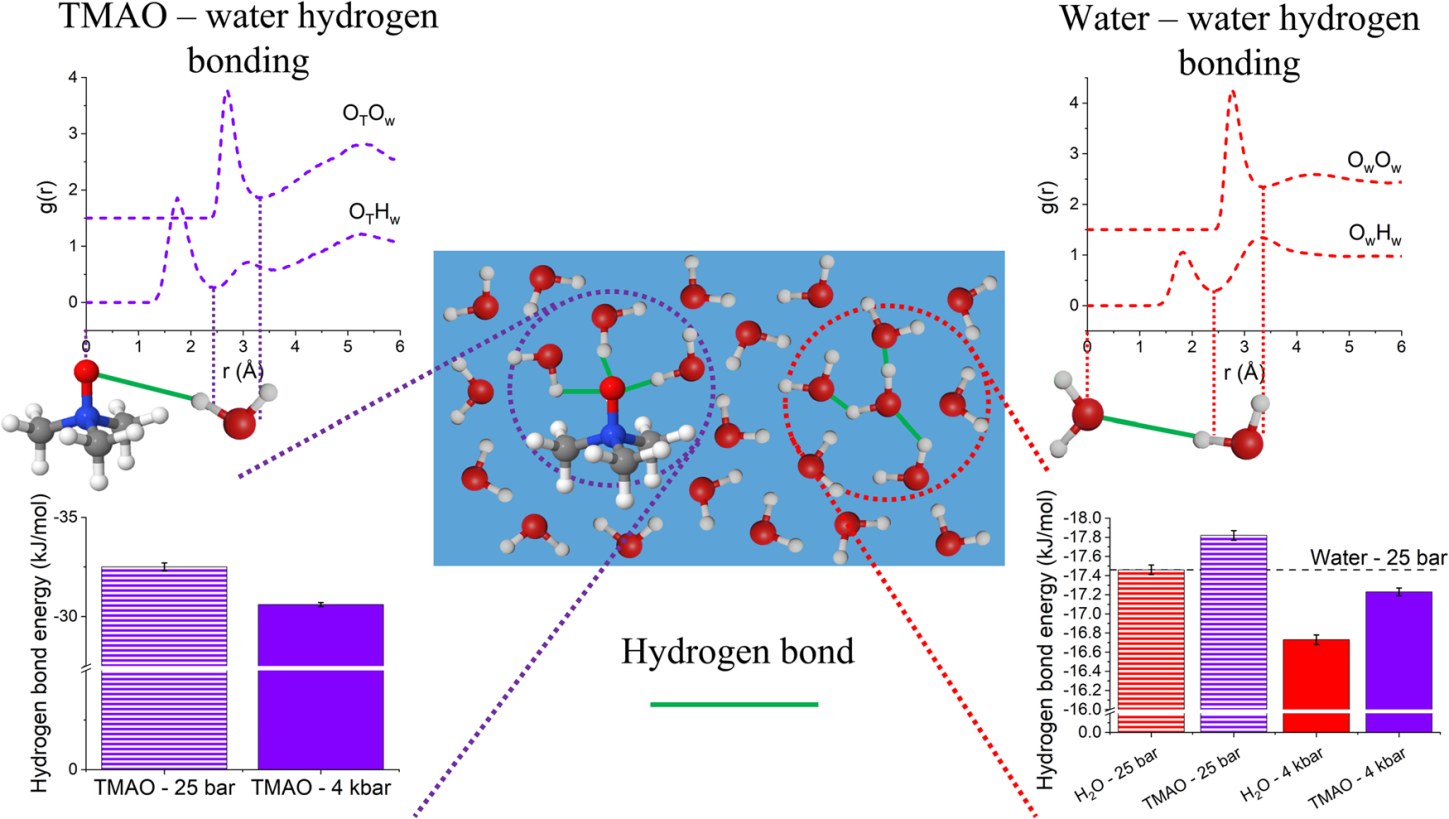
Hydrogen bonding in aqueous TMAO. A schematic showing the hydrogen bonding between water and a central TMAO oxygen (purple circle) and between water and a central water molecule (red circle) with the associated radial distribution functions, g(r), and calculated average hydrogen bond interaction energies.

The differences in water structure and energetics can now be considered in terms of perturbations to water–water hydrogen bonding from increased pressure and/or TMAO addition. A water molecule is donating a hydrogen bond to a central water molecule or TMAO oxygen if it simultaneously satisfies two criteria: its oxygen must be within a distance corresponding to the first minimum in the O_*w*_/O_*T*_O_*w*_ *g*(*r*), and hydrogen must be within a distance corresponding to the first minimum in the O_*w*_/O_*T*_H_*w*_ *g*(*r*). Only water molecules that are not within the first hydration shell of a TMAO molecule are allowed to be considered central water molecules. This distinction is made to determine whether the effects of TMAO extend into the bulk solvent. This is shown visually in Fig. 5, and the cutoff distances used in this work are described in Table S7 in the supplementary information. Once this condition is satisfied, the hydrogen bond interaction energy can be calculated according to the total Lennard-Jones and Coulomb potential between each pair of atoms in the hydrogen-bonded molecules according to the EPSR reference potentials described in Table S3 in the supplementary information. The calculated water–water hydrogen bond energy distributions are reported in Fig. S10 in the supplementary information with fitting parameters given in Tables S8 and S9 and further discussion in note S3. Upon increasing pressure from 25 bar to 4 kbar, the average water–water hydrogen bond interaction energy becomes less stable, increasing from −17.46 ± 0.05 kJ mol^−1^ to −16.73 ± 0.05 kJ mol^−1^. Upon the addition of TMAO at 2.0 mol kg^−1^ H_2_O, the average water–water hydrogen bond becomes more stable relative to pure water at equivalent pressure, determined to be −17.82 ± 0.05 kJ mol^−1^ and −17.23 ± 0.04 kJ mol^−1^ at 25 bar and 4 kbar, respectively. This clearly demonstrates the ability of TMAO to resist the pressure-induced structural perturbation to water. It is also consistent with results which indicate TMAO enhances hydrogen bonding between water molecules that have been reported in the previous literature^34,38,48,53,57,58,71^.

Next, we consider the molecular origins of the ability of TMAO to resist the pressured induced perturbations to water structure. We first consider hydrogen bonding between TMAO and water molecules. From the calculation of the TMAO–water bonding energies (Fig. 5), it is clear that the bonding is much stronger than water–water hydrogen bonding, measured to be −32.5 ± 0.2 kJ mol^−1^ at 25 bar and −30.6 ± 0.1 kJ mol^−1^ at 4 kbar. The distributions of TMAO–water hydrogen bond interaction energies, the average values in each case and the fitting parameters with associated uncertainties are given in the supplementary information (Fig. S11, Tables S10 and S11, and note S4). This reduction in stability upon increasing pressure is consistent with the results of Kolling et al.^72^, most likely due to a slightly distorted hydration shell required to accommodate more water molecules.

Finally, we consider the hydrogen bonding interaction energy between a central water molecule that does lie within the hydration shell of TMAO and a neighbouring water molecule. This contrasts with the previous measurements of water–water hydrogen bonding which only considered water molecules that did not lie within the first hydration shell of a TMAO molecule. In this case, a central water molecule can lie within the first hydration shell of the TMAO oxygen, referred to as hydrophilic water, or a TMAO methyl group, referred to as hydrophobic water. We calculate that for aqueous TMAO at 25 bar, hydrophilic water–water hydrogen bonds are more stable compared with the bulk water–water hydrogen bonds, determined to be −18.4 ± 0.1 kJ mol^−1^ and −17.82 ± 0.05 kJ mol^−1^, respectively. Somewhat surprisingly, the same is true for hydrophobic water–water hydrogen bonds, calculated to be −17.95 ± 0.07 kJ mol^−1^. As pressure is increased to 4 kbar, and TMAO–water hydrogen bonding is weakened, we calculate that hydrophilic water–water hydrogen bonding is also weakened, determined to be −17.48 ± 0.08 kJ mol^−1^, yet remains stronger than bulk water–water hydrogen bonding, determined to be −17.23 ± 0.04 kJ mol^−1^. Again, the same is true for hydrophobic water–water hydrogen bonding, which becomes less table upon increasing pressure, increasing from −17.95 ± 0.07 kJ mol^−1^ to −17.59 ± 0.06 kJ mol^−1^, but remaining stronger than the bulk water–water hydrogen bonds. The calculated water–water hydrogen bond energy distributions are reported in Fig. S12 in the supplementary information.

## Discussion

The O_*w*_O_*w*_ *g*(*r*) presented in Fig. 2 clearly demonstrate the resisting ability of TMAO, as the position of the peaks in the *g*(*r*) are less sensitive to pressure in aqueous TMAO than they are in pure water, in particular in retaining a clear second solvation shell. If we consider the concentration of TMAO in the muscle tissue of teleosts presented in Fig. 1, the trendline indicates TMAO at 2.0 mol kg^−1^ H_2_O should resist a pressure of ~3 kbar (see supplementary information Fig. S13 and note S5). While TMAO addition at 4 kbar aids in retaining a second O_*w*_O_*w*_ solvation shell, the overall perturbation to the O_*w*_O_*w*_ $g\left(r\right)$ is fairly minimal. We therefore suggest that the ability of TMAO to preserve O_*w*_O_*w*_ structure against external pressure is not the most significant driving force behind its previously observed pressure-resisting ability. However, the calculated average water–water hydrogen bond interaction energies are presented in Fig. 5 and show that, in the presence of TMAO, water–water hydrogen bonding is more stable. It is important to note here that the reported average values are calculated by considering the hydrogen bond interaction energy between every appropriately positioned pair of water molecules in the simulation box over several well-separated Monte Carlo iterations. The results of this method produce a distribution of hydrogen bond energies that is strongly reminiscent of a Gaussian distribution, hence the reported value is the location of the distribution peak with associated uncertainty. Only water molecules that do not lie within the first hydration shell of a TMAO molecule are considered in this measure, hence any heterogeneity in the water network structure arising from strongly contrasting hydration structures around the hydrophobic/hydrophilic regions of the TMAO molecule does not affect the reported values. They therefore reflect global perturbations to the water network, rather than a weighted average of an unperturbed bulk water structure and a strongly perturbed TMAO hydration water structure.

When we do consider the water molecules that lie within TMAO hydrophilic or hydrophobic hydration shell, we observe that in aqueous TMAO at 25 bar and 4 kbar hydrophilic water–water hydrogen bonds and hydrophobic water–water hydrogen bonds are stronger than bulk water–water hydrogen bonds. This suggests that both hydrophilic and hydrophobic hydration are of key importance to TMAO’s ability to act as a biomolecular stabilising agent. This is in excellent agreement with the DFT calculations of Miranda-Quintana et al.^39^, who consider the stabilising co-solutes TMAO, betaine, ectoine, and glycine. These results demonstrate that stabilisers tend to have large dipole moments and therefore increased hydrophilic character. They also show that a higher density of hydrophobic groups in the molecular structure of the co-solutes (TMAO > betaine > ectoine > glycine) yields greater stabilisation. This observation is also consistent with the “law of matching water affinities” proposed by Collins^69,77^. Protein surfaces, by definition, must be more hydrophilic than their hydrophobic core. Osmolytes with large hydrophobic character are therefore more likely to be excluded from the protein surface and promote biomolecular stability. We therefore propose that stabilising osmolytes require both large hydrophobic character to promote stabilisation of the surrounding water network and preferential exclusion and hydrophilic character to promote stabilisation of the surrounding water network and allow them to be sufficiently soluble. In this respect, TMAO is ideal.

**Fig. 6:**
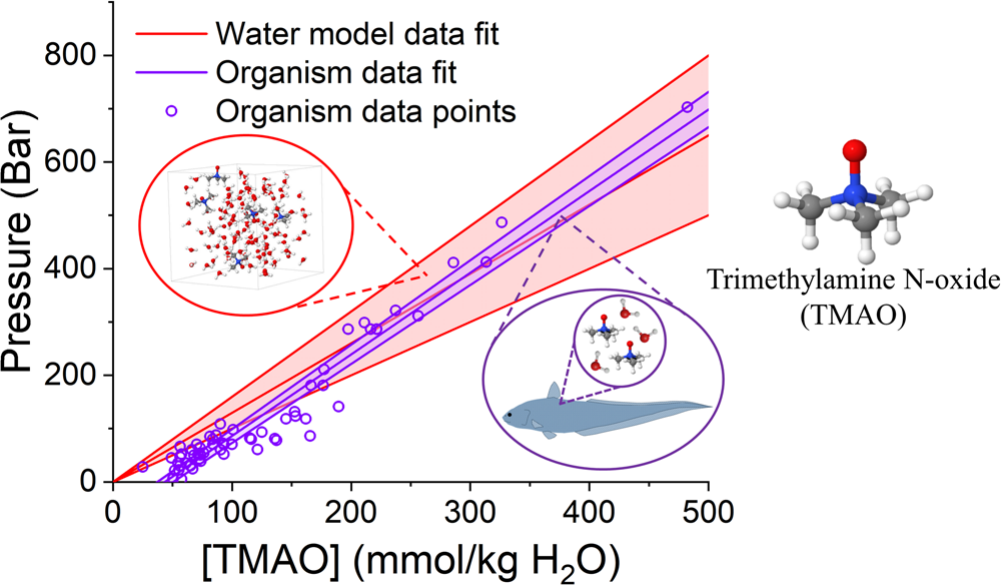
Agreement between model system and complete organisms. The pressure-resisting ability of TMAO as calculated by considering the perturbation to water − water hydrogen bonding by TMAO addition and pressure (red) through neutron scattering ($P=1.3\left(\pm 0.3\right)\times \left[{{{{{{\mathrm{TMAO}}}}}}}\right]$). This is compared with data reported by Yancey et al. (purple) on the concentration of TMAO in the muscle tissue of teleosts and the pressure at which they were harvested^26^ ($P=1.53\left(\pm 0.05\right)\times \left[{{{{{{\mathrm{TMAO}}}}}}}\right]-67\left(\pm 8\right)$).

We finally consider how the highly simplified system investigated in this work compares with much more complex biological organisms. With increased external pressure, the strength of water–water hydrogen bonding is measured to be less stable. If we first consider the rate of change of water–water hydrogen bond interaction energy with respect to pressure at constant TMAO concentration (pure water and 2.0 mol kg^−1^ H_2_O), we determine that in pure water, the average water–water hydrogen bond interaction energy becomes less negative at a rate of 0.17 ± 0.02 kJ mol^−1^ kbar^−1^. With respect to TMAO concentration at constant pressure, we calculate that TMAO addition causes water–water hydrogen bond interaction energy to become more negative at a rate of −0.22 ± 0.04 kJ mol^−1^(mol kg^−1^ H_2_O). Given the two different rates, the pressure-resisting ability of TMAO can be expressed in terms of the relative rate of change of water hydrogen bonding energies for TMAO and pressure by the ratio 1 : 1.3 ± 0.3 (mol kg^−1^ kbar^−1^), that is, TMAO at 1.0 mol kg^−1^ H_2_O resists a pressure of 1.3 ± 0.3 kbar. This can be plotted as a function of TMAO concentration, as shown by the red line and error ribbon in Fig. 6.

In order to compare this observed ratio of pressure-resisting ability of TMAO in water with what happens in actual living organisms, we have adapted the data of Yancey et al.^26^ (as reported here in Fig. 1) by converting sea depth to equivalent water pressure as derived in note S5 of the supplementary information. These data are shown as the purple dots and guidelines in Fig. 6. From this graph, it is clear that, although there may well be non-linear effects in the real organisms (which we are not sensitive to in the present work), the size of the pressure-resisting capability of TMAO in water is completely consistent with the concentration of TMAO in muscle tissue as a function of sea depth observed in nature.

This suggests that, quite generally, the teleost fish accumulate TMAO at an appropriate concentration to correctly compensate for the destabilising effects of pressure on water–water hydrogen bonding. Preserved water–water hydrogen bonding will, in turn, help to preserve crucial biomolecule–water hydrogen bonding and hydrophobic interactions against increased pressure, inhibiting the shifted thermodynamic equilibrium that results in more water molecules occupying internal cavities leading to denaturation^19,34,78^. We, therefore, demonstrate the apparent universality of the relation that perturbations at the molecular level due to piezolyte addition and pressure variation directly influence the ability of the complete organism to thrive in its habitat.

## Conclusion

In this work, we have used neutron scattering coupled with computational modelling to clearly demonstrate that TMAO is capable of resisting the pressure-induced structural perturbation to water. TMAO is shown to stabilise water–water hydrogen bonding against the destabilising effects of increased external pressure. We determine that the pressure-resisting ability of TMAO can be expressed by the ratio 1 : 1.3 ± 0.3 (concentration TMAO in mol kg^−1^ H_2_O : pressure resisted in kbar). This ratio is in good agreement with experimental data on the concentration of TMAO in the muscle tissue of teleosts harvested from different pressures. We propose that the origin of this effect is due to the hydration of the TMAO molecule itself. The negatively charged solvent exposed TMAO oxygen can form strong hydrogen bonds with the surrounding water molecules, resulting in a less compressible hydration shell around the oxygen, and the weakly interacting TMAO methyl group does not promote tetrahedral ordering of the surrounding water, also resulting in a less compressible hydration shell. We also demonstrate water–water hydrogen bonds are stronger in the hydration shell of both the hydrophobic methyl groups and hydrophilic oxygen compared within the bulk liquid at both pressures. TMAO can therefore be thought of as providing a structural anchor within the aqueous system, from which water can build a more stable network which extends into the bulk liquid and resist pressure-induced structural perturbations. These findings have important implications for the study of pressure-adapted extremophiles, which use TMAO to stabilise their biochemistry against the detrimental effects of increased pressure, the role of protecting osmolytes in phase transition kinetics in proteins^79^, as well as the study of the mechanism of how osmolytes protect in general.

## Methods

### Neutron scattering

Experimental neutron scattering data were obtained at 20 °C on the Near and InterMediate Range Order Diffractometer (NIMROD) instrument at the ISIS pulsed neutron and muon source, UK, covering a scattering vector *Q* range of 0.02–50 Å^80^ (experiment RB1910455, DOI: 10.5286/ISIS.E.RB1910455). The raw data were corrected for multiple scattering, attenuation, and inelastic scattering effects using Gudrun software^81^, resulting in the total structure factor *F*(*Q*). The resulting *F*(*Q*) can be expressed in the form shown in Eq. 1, where *c*_*α*_ is the concentration of atomic species *α*, *b*_*α*_ is the coherent neutron scattering length of atomic species *α,* which is dependent on the isotope species, and *S*_*αβ*_(*Q*) is the partial structure factor between atomic species *α* and *β*.

1 $$F\left(Q\right)=\mathop{\sum}\limits_{\alpha \beta }{c}_{\alpha }{c}_{\beta }{b}_{\alpha }{b}_{\beta }\left({S}_{\alpha \beta }\left(Q\right)-1\right)$$

*S*_*αβ*_(*Q*) can be related to the radial distribution function *g*_*αβ*_(*r*) by Fourier transform shown in Eq. 2. The integral of *g*_*αβ*_(*r*) between distances *r*_1_ and *r*_2_ therefore corresponds to the number of atoms *β* located between *r*_1_ and *r*_2_ from *α*, or the coordination number.

2 $${S}_{\alpha \beta }\left(Q\right)=\rho {\int }_{0}^{{{\infty }}}{g}_{\alpha \beta }\left(r\right){\exp }\left({iQr}\right){dr}=4\pi \rho {\int }_{0}^{{{\infty }}}{g}_{\alpha \beta }\left(r\right)\frac{{\sin }{Qr}}{{Qr}}{dr}$$

### Sample preparation

We employ hydrogen/deuterium substitution, as these isotopes have very different coherent scattering lengths (*b*_*H*_ = −3.74 fm and *b*_*D*_ = 6.67 fm)^82^. Milli Q pure water was used for protinated water, and deuterated water was provided by the ISIS facility. Protinated anhydrous TMAO was purchased from Sigma–Aldrich Merck and deuterated anhydrous TMAO was purchased from CK isotopes. Both were used without further purification. A complete sample list is given in the supplementary information (note S6 and Table S12).

All samples were investigated at two pressures: 25 bar and 4 kbar. The low 25 bar pressure was chosen to be sufficiently high such that the high-pressure cell (pressure applied through a mechanical piston provided by the ISIS facility^83^) was clearly in contact with the sample but sufficiently low such that structural perturbations to the solutions were minimal. The high 4 kbar pressure was chosen to be sufficiently high such that a clear perturbation to water structure would be evident through the *g*(*r*)s but not so high that any potential counteracting effects of TMAO would be overwhelmed^64^.

Aqueous samples containing TMAO were all studied at a TMAO concentration of 2.0 mol kg^−1^ H_2_O. This concentration was chosen based on the data presented in Fig. 1 (Yancey et al. (2014)) originally reported by Yancey et al.^26^. We use this data to estimate the pressure-resisting ability of TMAO as a function of TMAO concentration. The predicted relationship derived in the supplementary information (note S5 and Fig. S13) states that a TMAO concentration of 2.0 mol kg^−1^ H_2_O should resist a pressure of ~3.0 kbar. At 2.0 mol kg^−1^ H_2_O the pressure-resisting ability of TMAO should therefore be evident in the water–water *g*(*r*)s.

### EPSR simulations

EPSR is a Monte Carlo simulation-based structure refinement technique^73^. Within EPSR a box of molecules with periodic boundary conditions is built at the same concentration and density as the experimental samples. Each atom within the box is then described by a “reference” potential consisting of a charge *q* and the two Lennard-Jones parameters *ε* and *σ*. A second “empirical” potential is included, which is derived from the difference between the supplied experimental scattering data and the predicted scattering data from the simulation. The result is a simulated box of molecules whose scattering pattern is consistent with and constrained by the supplied diffraction data from several isotopically distinct samples, the system density, known molecular structures and is based on reasonable starting parameters for the energetics of the system. This technique has been successfully applied to various aqueous solutions and ionic liquids^51,56,57,63,84–94^.

For this study, a box size of 5000 water molecules was used for pure water samples, and a box size of 5550 water molecules and 200 TMAO water molecules was used for aqueous TMAO samples. The densities of the experimental samples were taken from previous literature^50,95–97^ and are outlined in table S12 in the supplementary information, along with simulation box dimensions. Values for the reference potential for water were taken from the extended simple point charge potential. This has been shown to accurately model the structure and dynamics of water^98^ and has been successfully employed in various previous EPSR studies^56,57,70,76,85,91,92,99,100^. Two different reference potentials published by Meersman et al.^51,52^ and Hölzl et al.^47^ were used to describe TMAO to help test the sensitivity of the final simulations to the starting reference potential. The results using the Meersman potential are discussed here, and those using the Hölzl potential are described in the supplementary information. These reference potentials, along with the final fits to the experimental data, are reported in table S3 and Fig. S14 in the supplementary information.

### Extended analysis routines

We attempt to monitor water–water hydrogen bond interaction energies, TMAO–water hydrogen bond interaction energies, water–water dipole angle distributions, TMAO oxygen–water dipole angle distributions, and TMAO methyl–water dipole angle distributions through a custom analysis routine^56,57,74^.

This analysis routine first reads in the coordinates for all simulated atoms produced through ESPR. Hydration and edge water molecules are then distinguished from bulk water molecules. Hydration water molecules are defined as water molecules whose oxygen lies within 3.38 Å from a TMAO oxygen or 4.48 Å from a TMAO carbon. These cutoff distances correspond to the location of the first minimum in the associated *g*(*r*)s for aqueous TMAO at 25 bar. Edge water molecules are defined as water molecules whose oxygen lies within 3.38 Å from the edge of the simulation box, corresponding to the location of the first minimum in the water oxygen (O_*w*_–O_*w*_) *g*(*r*) for pure water at 25 bar. The remaining water molecules are then classified as bulk water molecules. Only these molecules are considered central molecules in the calculation of water–water hydrogen bonding or dipole angles.

Hydrogen bond interaction energies between a central water molecule or TMAO oxygen and a hydrating water molecule, and the hydrating water dipole angle around a central atomic species, are then calculated. The interaction energy is calculated using the sum of the Coulomb potential and the Lennard-Jones potential between the two hydrogen-bonded molecules according to the EPSR reference potentials. The results are then binned with bin widths of 1 kJ mol^−1^ and normalised to the total number of detected hydrogen bonds.

## Supplementary information

Supplementary Information

## Data Availability

Raw neutron scattering data are available from: 10.5286/ISIS.E.RB1910455. Raw and processed data presented in Figs. 2–6 are available from: 10.5518/1125. Python scripts required for data analysis included.

## Code availability

Python scripts required for data analysis can be found at: 10.5518/1125.
