# Supplementary material for: The ability of trimethylamine N-oxide to resist pressure induced perturbations to water structure
Source: Commun Chem. 2022 Sep 28;5:116. doi: 10.1038/s42004-022-00726-z (PMC9814673; doi:10.1038/s42004-022-00726-z)
Supplement: Supplementary file 1 — Supplementary Information [file 42004_2022_726_MOESM1_ESM.docx]

# Supplementary Information

The Ability of Trimethylamine N-Oxide to Resist Pressure Induced Perturbations to Water Structure

Harrison Laurent, Tristan G. A. Youngs, Thomas F. Headen, Alan K. Soper, Lorna Dougan


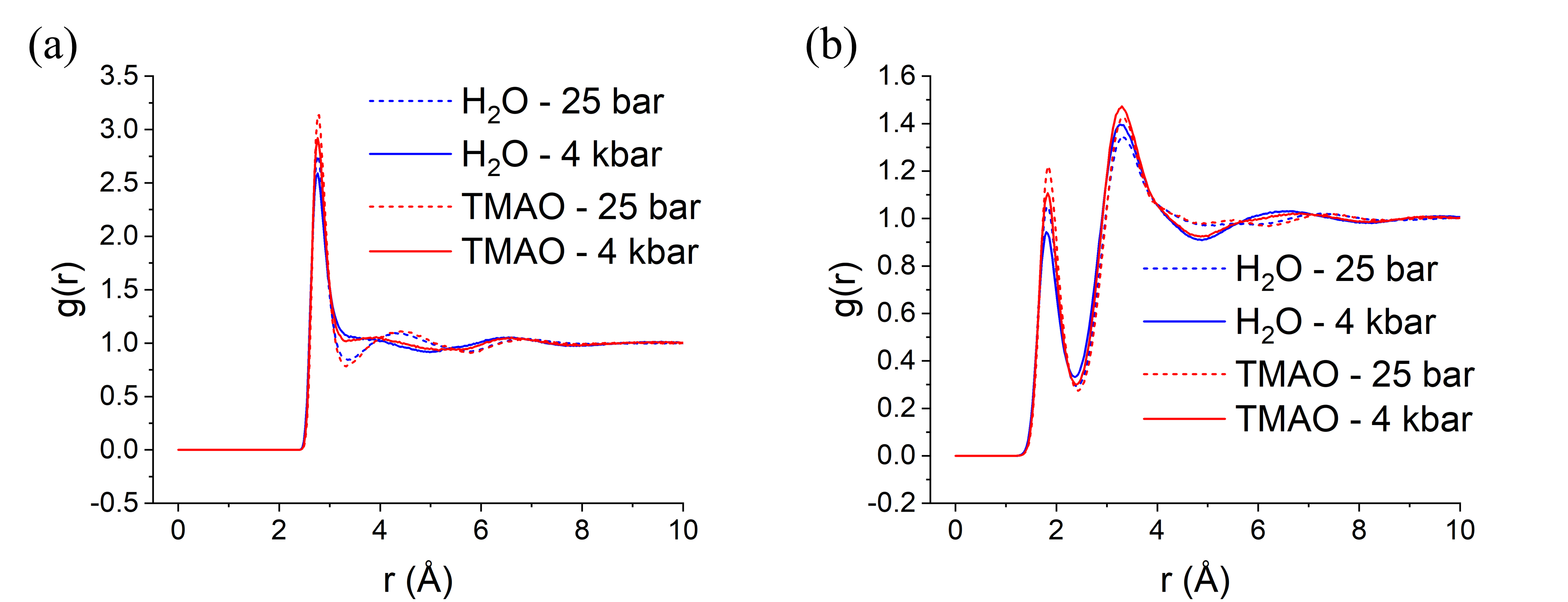


**Figure S1:** O*_w_*O*_w_* (a) and O*_w_*H*_w_* (b) *g*(*r*)*s* for pure water (blue) and aqueous TMAO (red) at 25 bar (dashed) and 4 kbar (solid).

**Table S1:** Peak positions of the first and second peaks in the O*_w_*O*_w_* RDFs for pure water and aqueous TMAO at 2.0 mol/kg H_2_O at 25 bar and 4 kbar calculated through EPSR.

| System | First peak (Å) | Second peak (Å) |
| --- | --- | --- |
| H_2_O at 25 bar | 2.77 | 4.35 |
| H_2_O at 4 kbar | 2.76 | N/A |
| Aqueous TMAO at 25 bar | 2.78 | 4.42 |
| Aqueous TMAO at 4 kbar | 2.77 | 3.85 |


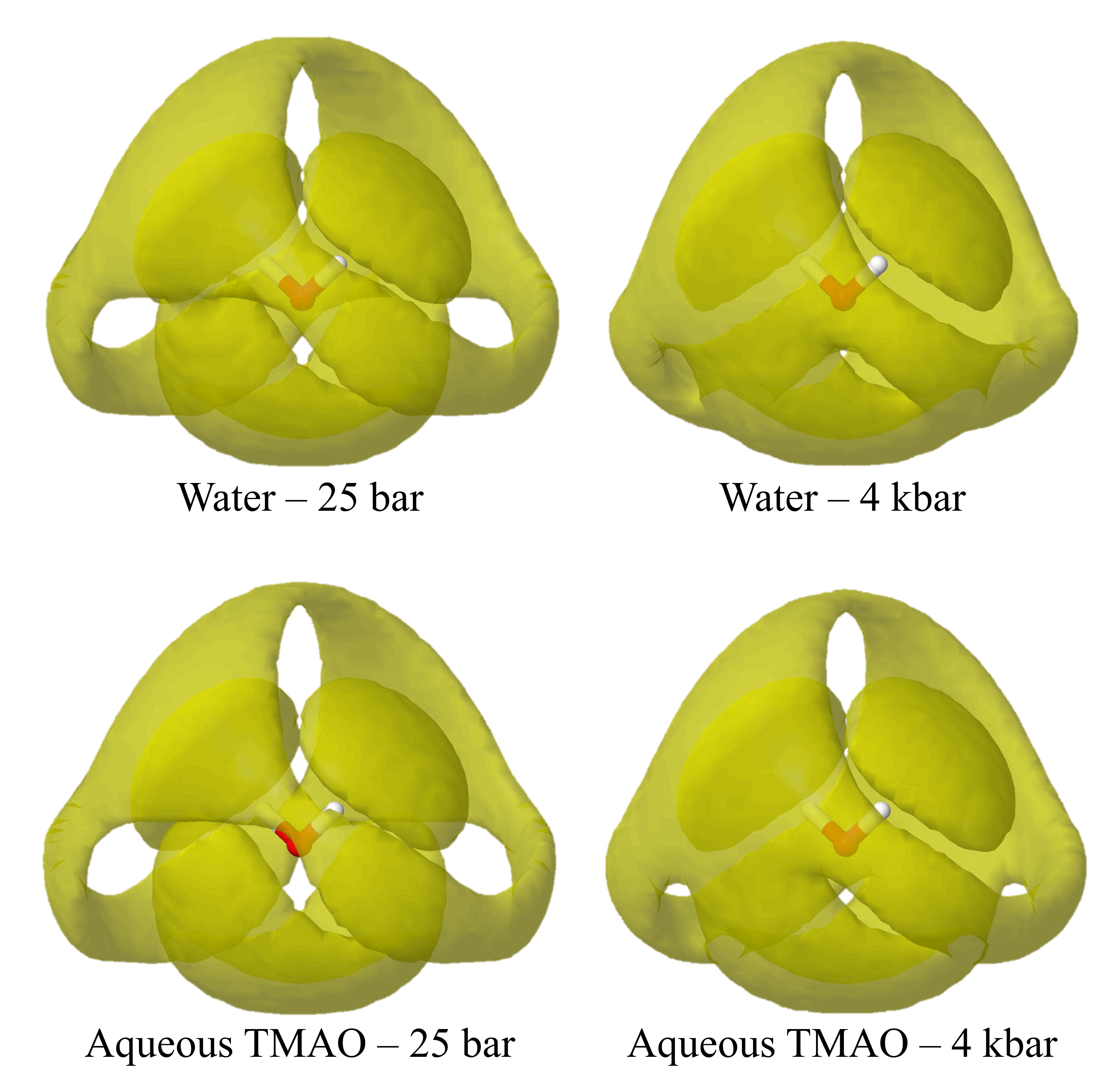


**Figure S2:** The spatial density functions for water and aqueous TMAO at 2.0 mol/kg H_2_O at 25 bar and 4 kbar calculated through EPSR. The yellow isosurface contains the 30% most likely positions for neighbouring water molecules around a central water molecule.


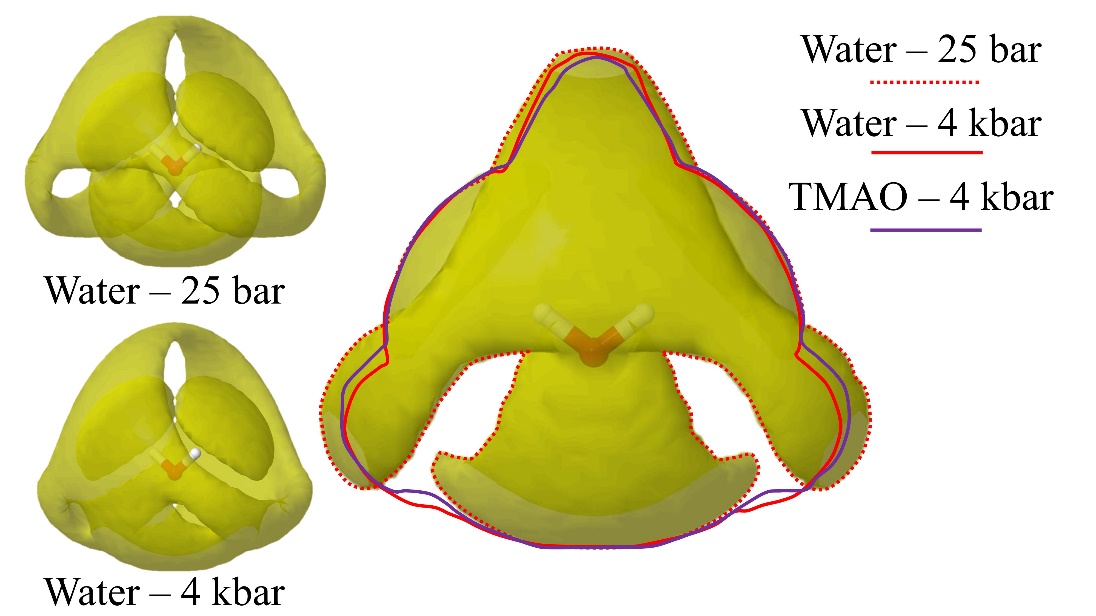


**Figure S3:** The water - water SDFs for pure water at 25 bar and 4 kbar as viewed from 35^◦^ normal to the plane of the central water molecule (left). The water - water SDF for pure water at 25 bar as viewed normal to the plane of the central water molecule. This SDF is outlined (red dashed) and the outline of pure water at 4 kbar (solid red) and the outline of aqueous TMAO at 2.0 mol/kg H_2_O at 4 kbar (solid purple) are superimposed onto the image. These isosurfaces represent the 30% most probable areas of finding a neighbouring water molecule relative to a central water molecule.

**Table S2:** O*_w_*O*_w_* coordination numbers calculated over 3.38 Å by EPSR, corresponding to the first minimum in the O_w_O_w_ *g(r)* at 25 bar. Many statistics drawn from from *>* 1000 EPSR iterations mean that the coordination numbers can be determined with error values *<* 0*.*01 and are therefore not reported.

| System | O_w_O_w_ Coordination number |
| --- | --- |
| H_2_O at 25 bar | 4.54 |
| H_2_O at 4 kbar | 5.44 |
| Aqueous TMAO at 25 bar | 3.97 |
| Aqueous TMAO at 4 kbar | 4.64 |


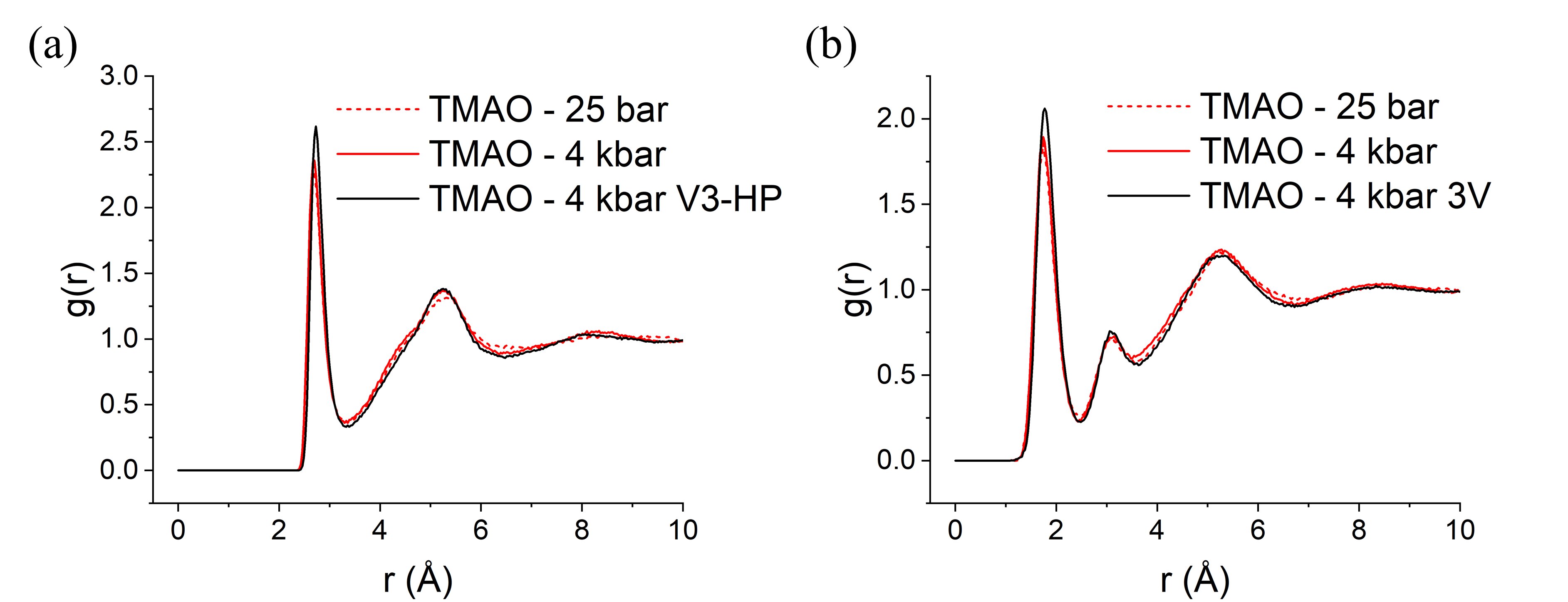


**Figure S4:** O*_T_*O*_w_* (a) and O*_T_*H*_w_* (b) *g*(*r*)*s* for aqueous TMAO at 25 bar (dashed) and 4 kbar (solid) using the Meersman potential (red) and the Hölzl V3-HP potential (black).


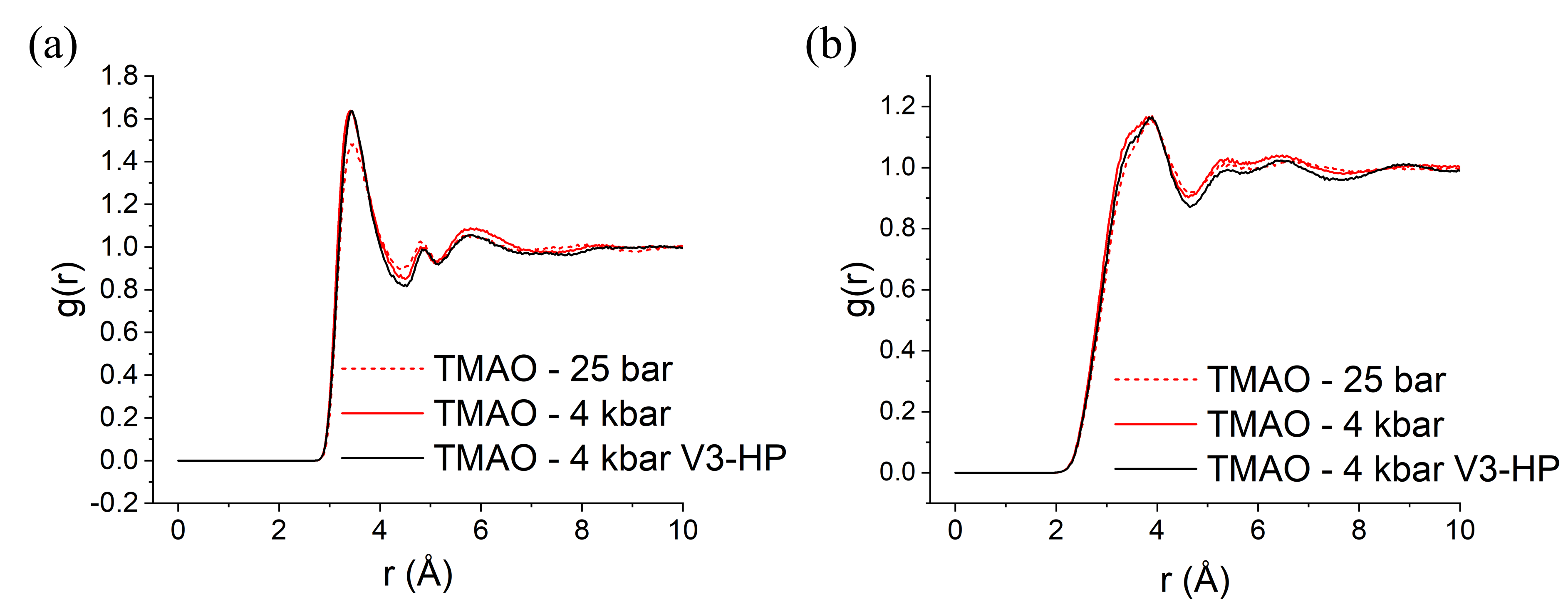


**Figure S5:** C*_T_*O*_w_* (a) and C*_T_*H*_w_* (b) *g*(*r*)*s* for aqueous TMAO at 25 bar (dashed) and 4 kbar (solid) using the Meersman potential (red) and the Hölzl V3-HP potential (black).

**Table S3:** The Lennard-Jones and coulomb parameters for aqueous TMAO. Values taken from^1^ and ^2^*.

| Atomic species | *ε* (kJ/mol) | *σ* (Å) | charge (e) |
| --- | --- | --- | --- |
| O*_w_* | 0.6500 | 3.166 | -0.8476 |
| H*_w_* | 0.0000 | 0.0000 | +0.4238 |
| C*_T_* | 0.3900 | 3.7000 | -0.2600 |
| H*_T_* | 0.0650 | 1.8000 | 0.1100 |
| N*_T_* | 0.7110 | 3.2500 | 0.4400 |
| O*_T_* | 0.5850 | 3.0800 | -0.6500 |
| C*_T_** | 0.2830 | 3.7070 | -0.2609 |
| H*_T_** | 0.7750 | 2.1300 | 0.1166 |
| N*_T_** | 0.8374 | 2.9260 | 0.5932 |
| O*_T_** | 0.6389 | 3.2660 | -0.8599 |

**Table S4:** O*_T_*O*_w_* coordination numbers calculated over 3.38 Å by EPSR. Many statistics drawn from from *>* 1000 EPSR iterations mean that the coordination numbers can be determined with error values *<* 0*.*01 and are therefore not reported.

| System | O*T*O*w*  number | coordination |
| --- | --- | --- |
| Aqueous TMAO at 25 bar | 2.65 |  |
| Aqueous TMAO at 4 kbar | 3.04 |  |
| Aqueous TMAO at 4 bar V3-HP | 3.09 |  |

**Table S5:** O*_T_*O*_w_* coordination numbers calculated over 4.48 Å by EPSR. Many statistics drawn from from *>* 1000 EPSR iterations mean that the coordination numbers can be determined with error values *<* 0*.*01 and are therefore not reported.

| System | C*T*O*w*  number | coordination |
| --- | --- | --- |
| Aqueous TMAO at 25 bar | 8.10 |  |
| Aqueous TMAO at 4 kbar | 9.44 |  |
| Aqueous TMAO at 4 bar V3-HP | 9.13 |  |

**Note S1:**

The dipole angle *θ* of a water molecule hydrating either a central water molecule, TMAO oxygen, or TMAO carbon, is defined as the angle between the vector which points from the central atom to the hydrating water molecule oxygen, and the vector corresponding to the hydrating water molecule dipole. The dipole vector on a water molecule is defined as the vector which points from the area of net negative charge to the area of net positive charge, and therefore originates at the water molecule oxygen and bisects the two hydrogens. This is shown visually in figure S6 in the supplementary information. This calculation is performed for every water molecule that is within the first hydration shell of a water molecule, TMAO carbon, or TMAO oxygen. These results are also binned with bin widths of 2° and normalised to the total number of data points so the final data represents the probability of finding a water molecule of dipole angle *θ*.


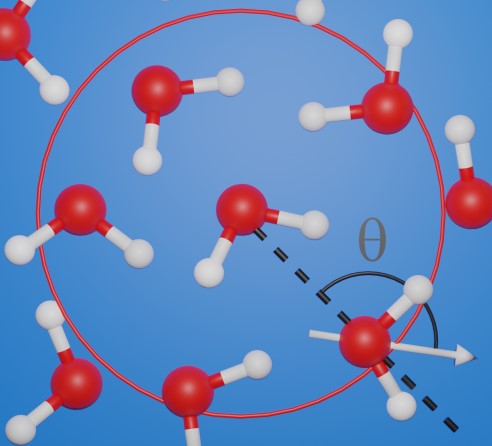


**Figure S6:** Definition of the dipole angle *θ* for a water molecule hydrating a central bulk water molecule. The width of the first hydration shell (defined as 3.38 Å in this work) is shown in red.

**Note S2:**

The water - water dipole angle distributions mirror the results of the water - water SDFs and O*_w_*O*_w_ g*(*r*)s. In pure water the water - water dipole angle distribution is shown to become less clearly defined as pressure is increased from 25 bar to 4 kbar, as indicated by reduced peak heights and a less shallow minimum between the two peaks. This definition is then regained upon the addition of TMAO at 2.0 mol/kg H_2_O at equivalent pressure. The water - water dipole angle distributions and the measured peak positions are shown in figure S9 and table S6. As this analysis only considers water molecules outside the first hydration shell of TMAO, this demonstrates that the perturbation to water structure by TMAO extends into the bulk solvent.

As O*_T_* is only capable of acting as a hydrogen bond acceptor, unlike a water molecule which can act as both donor and acceptor, the water dipole angle distribution only shows a single peak at low angles. In the case of aqueous TMAO at 25 bar this peak occurs at 49.4°. If one continues to use the Meersman forcefield, then upon increasing pressure from 25 bar to 4 kbar we observe that this peak shifts slightly outwards to 50.9° and becomes slightly broader, reflecting a slightly more poorly defined hydration structure. If one uses the Hölzl potential the peak width is essentially unchanged and the peak position occurs at 49.3°. In both instances we observe that similar to the O*_T_* - water *g*(*r*)s, the water dipole angle distribution around O*_T_* is far less sensitive to an external pressure than the bulk water structure.

In figure S8 we observe a broad distribution centred around 96° at both 25 bar and 4 kbar in the case of the Meersman potential, and 93° in the case of the Hölzl potential. When representing the dipole angle distributions in the manner used in this work, a complete lack of tendency for hydrating water molecules to orient their dipoles towards or away from a central entity would be represented by a single broad distribution centred at 90°. The distributions presented in figure S7 therefore represent a very weak tendency for water molecules to orient their dipole away from the slightly positively charged methyl groups, however the surrounding water molecules are essentially orientationally uncorrelated. This is in good agreement with the relatively featureless C*_T_*H*_w_ g*(*r*) displayed in figure S5(b).

The dipole angle distributions for water molecules in the first hydration shell of a central bulk water molecule are reported in figure S9. Here we observe a bimodal distribution of dipole angles in all cases. The first peak at lower angles reflects water molecules that are orienting their dipole towards the central bulk water molecule, and are therefore likely donating a hydrogen bond, and the second peak at larger angles reflects water molecules that are orienting their dipole away from the central bulk water molecule and are therefore likely accepting a hydrogen bond. In both pure water and aqueous TMAO at 2.0 mol/kg H_2_O the pressure induced structural perturbation to water is reflected by a reduction in height of both peaks and an increase in height of the minimum between the two peaks as one moves from 25 bar to 4 kbar. This is consistent with the more poorly defined water structure with increasing pressure indicated by the *g*(*r*)s and SDFs in section 3.1.


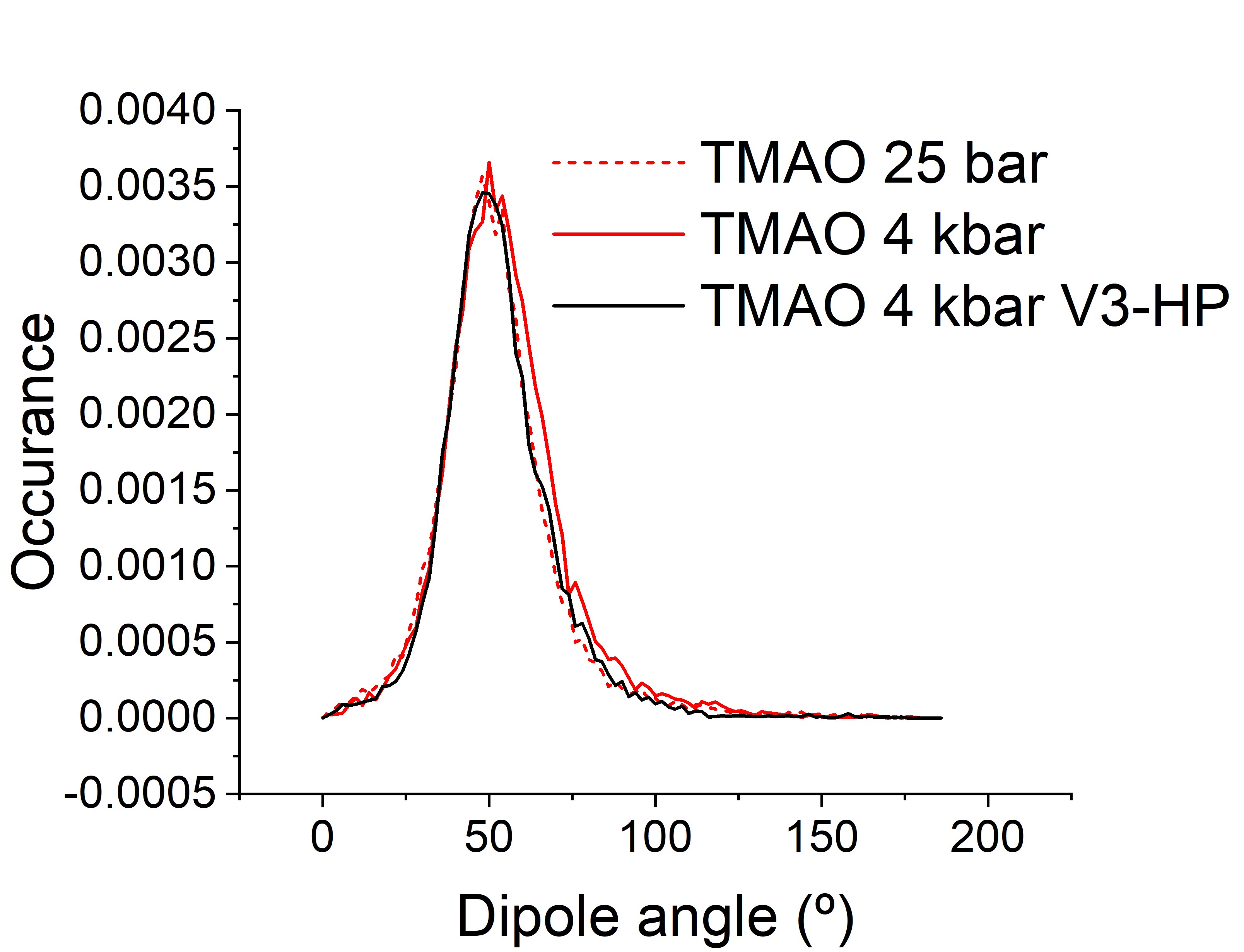


**Figure S7:** Water dipole angle distribution around a central TMAO oxygen in aqueous TMAO at 2.0 mol/kg H_2_O at 25 bar (dashed) and 4 kbar (solid) using the Meersman reference potential (red) and the Hölzl reference potential (black).


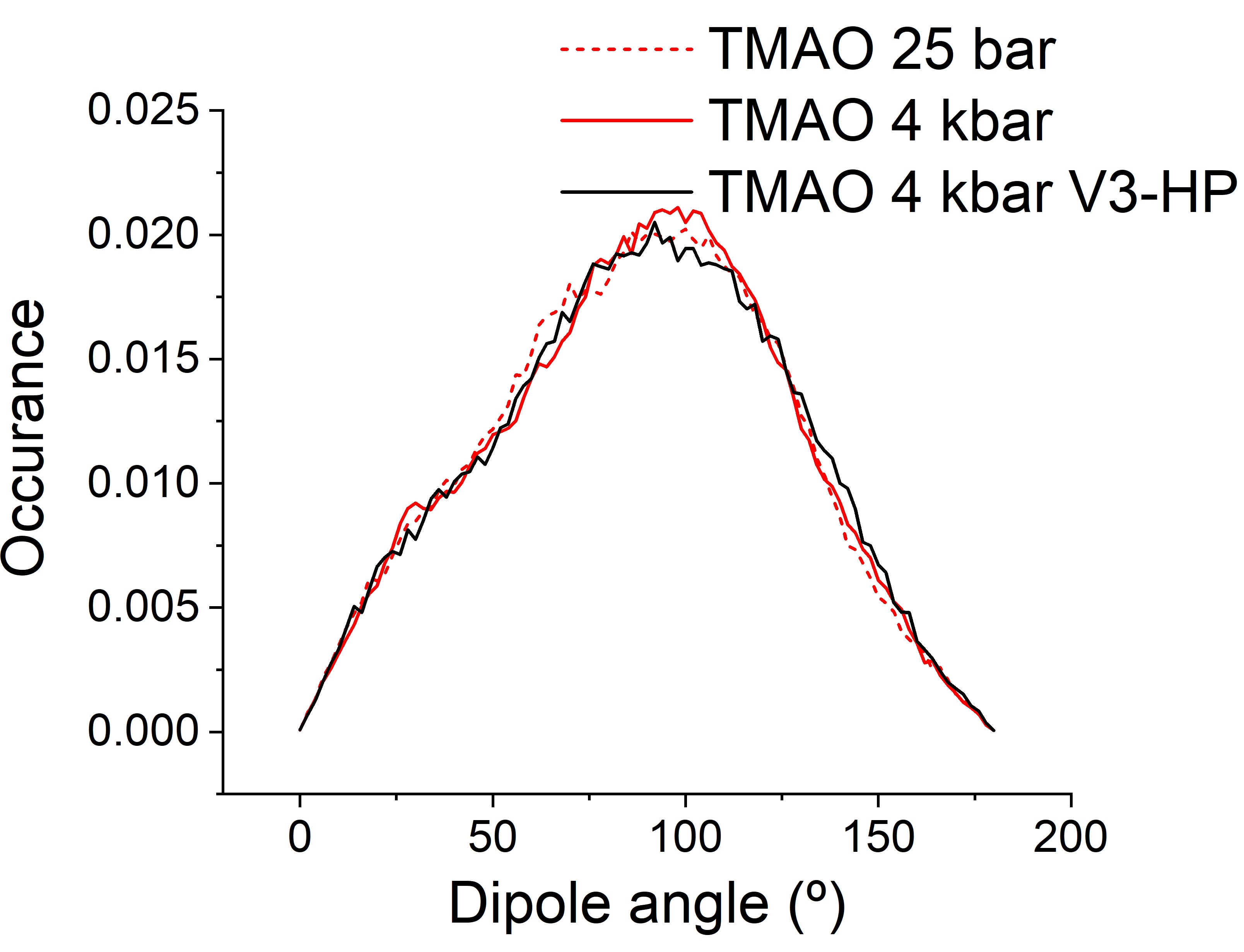


**Figure S8:** Water dipole angle distribution around a central TMAO methyl group in aqueous TMAO at 2.0 mol/kg H_2_O at 25 bar (dashed) and 4 kbar (solid) using the Meersman reference potential (red) and the Hölzl reference potential (black).


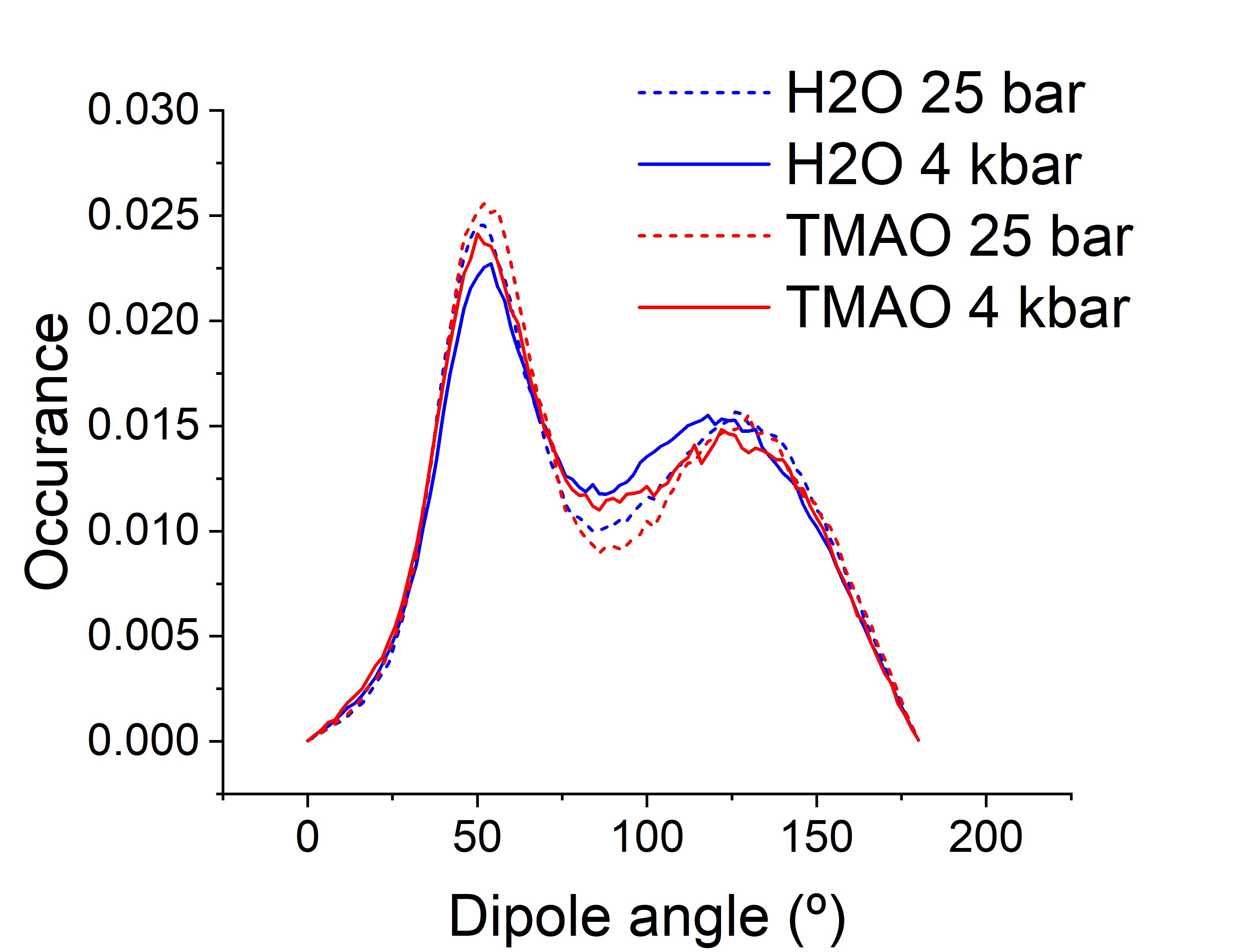


**Figure S9:** The dipole angle distribution for water molecules hydrating a central bulk water molecule.

**Table S6:** Peak positions of the first and second peaks in the bulk water water dipole angle distributions for pure water and aqueous TMAO at 2.0 mol/kg H_2_O at 25 bar and 4 kbar. The smooth distributions as a result of many statistics drawn from from 30 iterations of the analysis routine and large EPSR box sizes mean that the peak positions can be determined with error values *<* 0*.*1° and are therefore not reported.

| System | First peak (°) | Second peak (°) |
| --- | --- | --- |
| H_2_O at 25 bar | 50.9 | 127.2 |
| H_2_O at 4 kbar | 52.4 | 120.1 |
| Aqueous TMAO at 25 bar | 52.2 | 127.9 |
| Aqueous TMAO at 4 kbar | 51.6 | 123.1 |

**Table S7:** Cutoff distances used to define water - water hydrogen bonding and TMAO - water hydrogen bonding. Water - water hydrogen bonding cutoffs determined from the water - water *g*(*r*)s produced by EPSR of pure water at 25 bar and TMAO - water hydrogen bonding cutoffs determined from TMAO - water *g*(*r*)s produced by EPSR of aqueous TMAO at 25 bar.

| Atomic species | | Cutoff distance (Å) |
| --- | --- | --- |
| O*_w_* | - O*_w_* | 3.38 |
| O*_w_* | - H*_w_* | 2.41 |
| O*_T_* | - O*_w_* | 3.38 |
| O*_T_* | - H*_w_* | 2.48 |

**Note S3:**

The distributions of bulk water - water hydrogen bond interaction energies, calculated as described in the results section of the main text, are shown in figure S10(a). These distributions are well fit to a Gaussian distribution of form shown in equation S1 and fitting results are reported in table S8. The peak location and associated uncertainty calculated through this fitting are shown in figure S10(b) and table S9, and the remainder of the final fitting parameters with associated uncertainties and the quality of the fit measured by the $R^{2}$ value are given in table S8.

| $y=y_{0}+\frac{A}{w\sqrt{\frac{\pi}{2}}}\exp\left[ -2\left( \frac{x-x_{c}}{w} \right)^{2} \right]$ | (S1) |
| --- | --- |


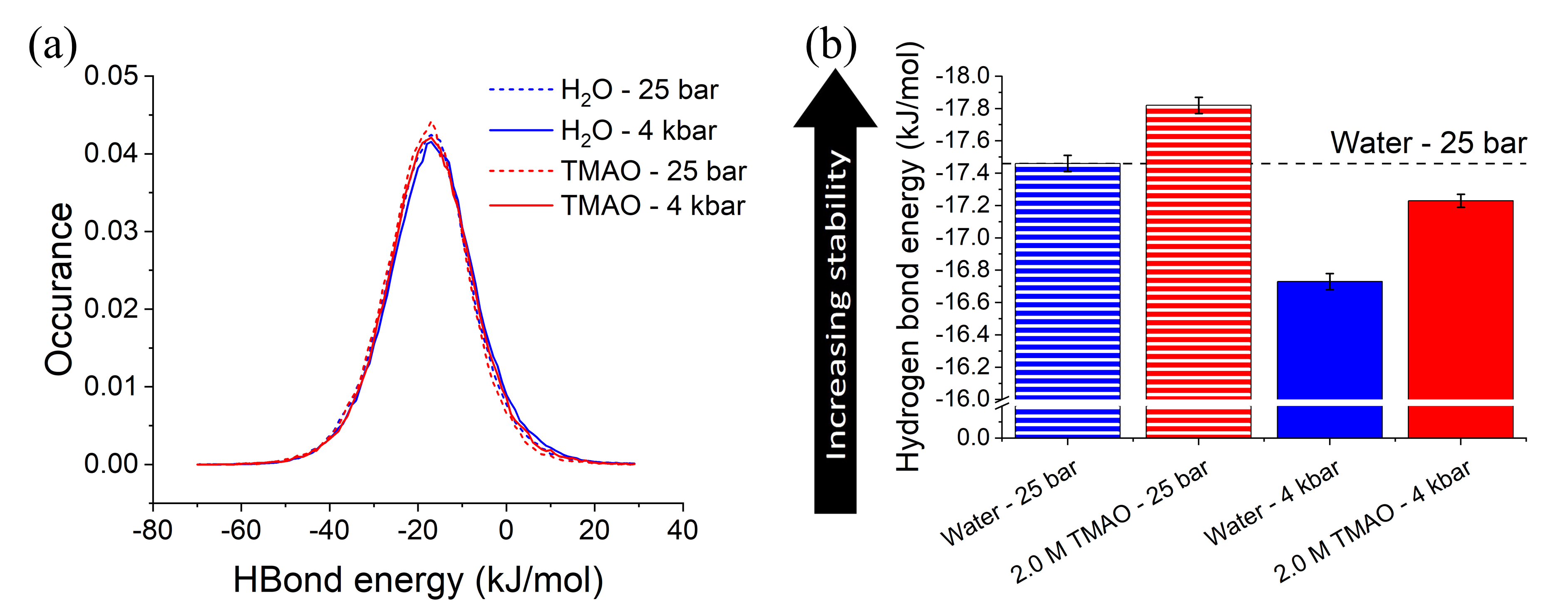


**Figure S10:** Bulk water - water hydrogen bond interaction energy distributions normalised to the total number of bulk water - water hydrogen bonds (a) and peak locations of distributions calculated according to equation S1 with associated uncertainty (b).

**Table S8:** Fitting parameters for gaussian distribution to bulk water – water hydrogen bond data. Occurrence shortened to occ in units.

| Fitting parameter | H_2_O at 25 bar | H_2_O at 4 kbar | Aqueous TMAO at 25 bar | Aqueous TMAO at 4 kbar |
| --- | --- | --- | --- | --- |
| $y_{0}\times{10}^{-4}$ (occ) | (5±1) | (5.5±0.9) | (4.6±0.9) | (4.8±0.7) |
| $x_{c}$ (kJ/mol) | -17.46±0.05 | -16.73±0.05 | -17.82±0.05 | -17.23±0.04 |
| $w$(kJ/mol) | 18.5±0.1 | 18.9±0.1 | 18.1±0.1 | 18.57±0.09 |
| $A$ (occ×kJ/mol) | 0.948±0.007 | 0.945±0.006 | 0.954±0.006 | 0.952±0.005 |
| $R^{2}$ | 0.998 | 0.998 | 0.998 | 0.999 |

**Table S9:** The peak positions in the bulk water - water hydrogen bond interaction energy distributions. Reported uncertainty is the uncertainty in the peak position calculated through performing a Gaussian fit to the raw distribution according to equation S1.

| System | Hydrogen Bond Interaction  Energy (kJ mol^−1^) |
| --- | --- |
| H_2_O at 25 bar | -17.46 ± 0.05 |
| H_2_O at 4 kbar | -16.73 ± 0.05 |
| Aqueous TMAO at 25 bar | -17.82 ± 0.05 |
| Aqueous TMAO at 4 kbar | -17.23 ± 0.04 |

**Note S4:**

If one uses the Hölzl potential to calculate the hydrogen bond interaction energies, then the average TMAO - water hydrogen bond interaction energy at 4 kbar decreases significantly, determined to be -39.7 ± 0.2 kJ mol^−1^. This stark difference between the hydration of O*_T_* if one alternates between the two force fields is not observed through the *g*(*r*)s reported in figure S4 or the dipole angle distributions reported in figure S7. This is therefore likely to be solely as a result of only using the EPSR reference potential when calculating hydrogen bond interaction energies, rather than using both the reference and empirical potentials. The increased dipole on the NO bond in the TMAO molecule present in the Hölzl potential means that a more negative interaction energy will be calculated for an identically structured system. The empirical potential is represented by a series of Poisson functions, which evolve with each EPSR iteration so as to drive the simulation to match the experimental scattering data. This constant evolution and their relatively complex form means that the inclusion of the empirical potential into the hydrogen bond interaction energy calculations is highly non-trivial. The observation that the *g*(*r*)s and dipole angle distributions are highly similar regardless of which potential is used suggests that the sum of the reference and empirical potential is very similar in both cases, and EPSR is functioning as it was intended. If one were to include the empirical potential in the calculation of the TMAO - water hydrogen bonds, it is therefore likely that the hydrogen bond energy distributions would be much more similar than reported in figure S11.


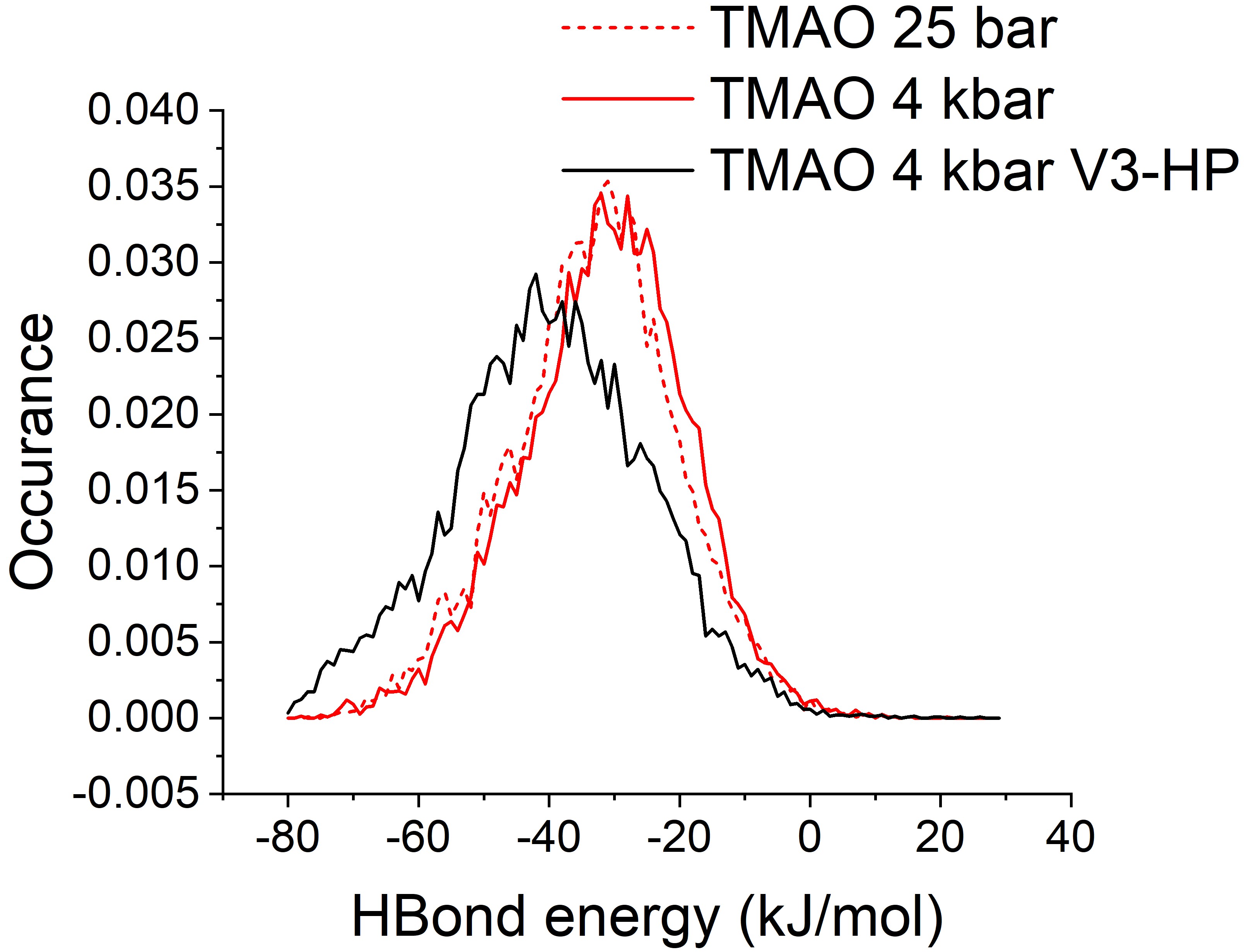


**Figure S11:** TMAO - water hydrogen bond energy calculated using the reference potential from EPSR in aqueous TMAO at 2.0 mol/kg H_2_O at 25 bar (dashed) and 4 kbar (solid) using the Meersman reference potential (red) and the Hölzl reference potential (black).

**Table S10:** Location of the peak in the TMAO - water hydrogen bond energy distributions. Reported uncertainty is the uncertainty in the peak position calculated through performing a Gaussian fit to the raw distribution.

| System | Peak location (kJ mol^−1^) |
| --- | --- |
| Aqueous TMAO at 25 bar | -32.5 ± 0.2 |
| Aqueous TMAO at 4 kbar | -30.6 ± 0.1 |
| Aqueous TMAO at 4 bar V3-HP | -39.7 ± 0.2 |

**Table S11:** Fitting parameters for gaussian distribution to TMAO – water hydrogen bond data with TMAO modelled with Meersman^1^ and Hölzl^2^ potential. Occurrence shortened to occ in units.

| Fitting parameter | Aqueous TMAO at 25 bar (Meersman) | Aqueous TMAO at 4 kbar (Meersman) | Aqueous TMAO at 4 kbar (Hölzl) |
| --- | --- | --- | --- |
| $y_{0}\times{10}^{-4}$ (occ) | 3±2 | 3±2 | 2±2 |
| $x_{c}$ (kJ/mol) | -32.5±0.2 | -30.6±0.1 | -39.7±0.2 |
| $w$(kJ/mol) | 24.0±0.4 | 24.1±0.3 | 29.7±0.5 |
| $A$ (occ×kJ/mol) | 0.96±0.02 | 0.97±0.01 | 0.99±0.02 |
| $R^{2}$ | 0.986 | 0.989 | 0.987 |

**
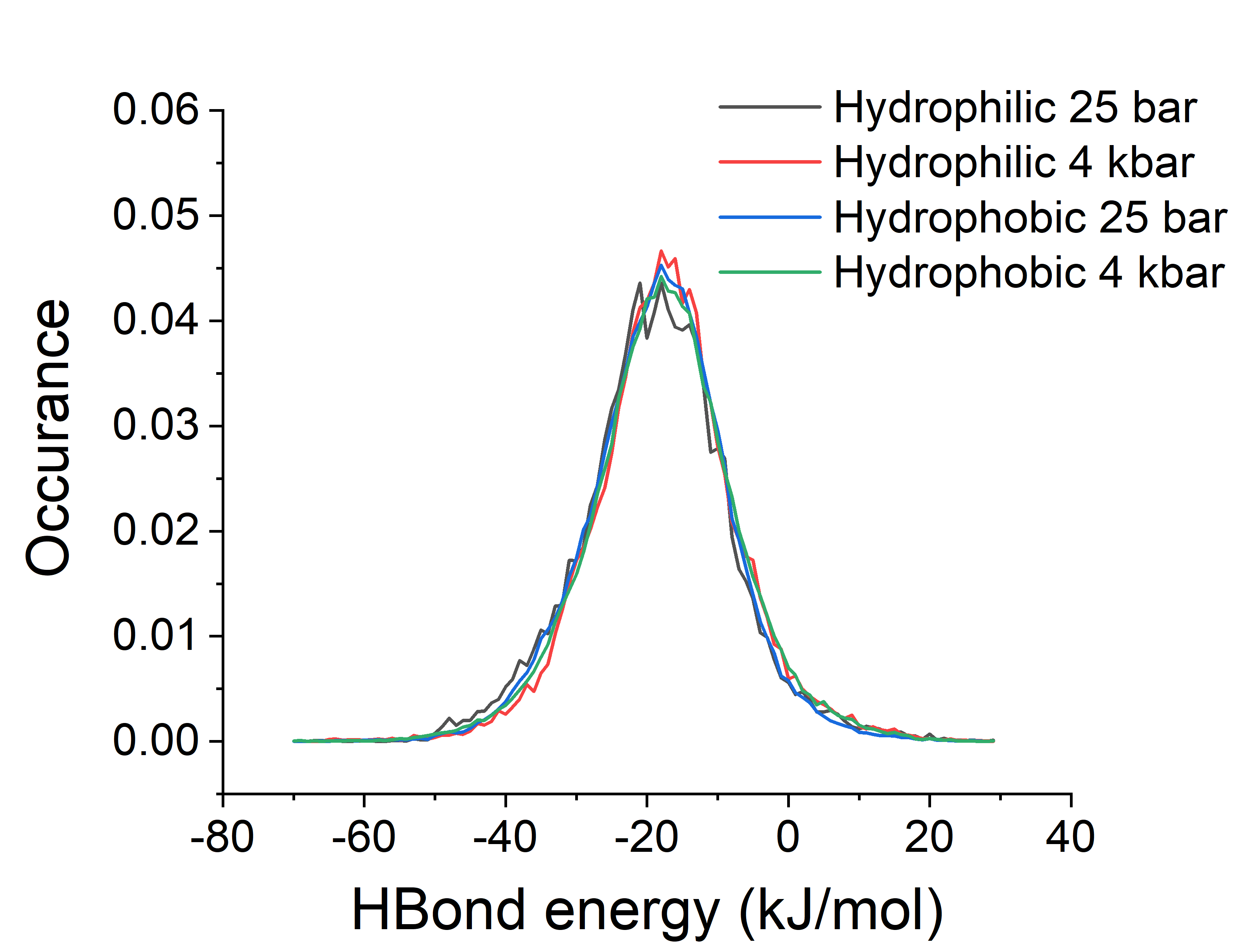
**

**Figure S12:** Hydrophilic and hydrophobic water - water hydrogen bond interaction energy distributions normalised to the total number of bulk water - water hydrogen bonds at 25 bar and 4 kbar

**Note S5: TMAO concentration vs resisted pressure derivation**

- TMAO concentration in mmol/kg wet muscle tissue as a function of depth from which organism was harvested for teleosts as reported by Yancey *et al* ^3^, Bockus *et al* ^4^, and Samerotte *et al* ^5^ in figure 1 of main text is replotted as pressure from which the organism is harvested as a function of TMAO concentration in mmol/kg H_2_O.
- Depth $d$ in meters is converted to pressure $P$ in bar by *P* = 0*.*1004 × *d*. TMAO concentration in mol/kg wet muscle tissue is then converted to mol/kg H_2_O by stating that teleost muscle tissue is roughly 80% water by mass^6^, hence $\left[ TMAO \right]{}_{{mol}/{kg} H_{2}O}=\frac{1}{0.8}\left[ TMAO \right]{}_{{mol}/{kg} wet muscle tissue}$
- Data replotted in figure S13
- Straight line fit to data predicts the relationship *P* = 1*.*53(±0*.*05) × [*TMAO*] − 67(±8)
- Using this relationship a TMAO concentration of 2.0 mol/kg H_2_O corresponds to a pressure of 3.0 kbar


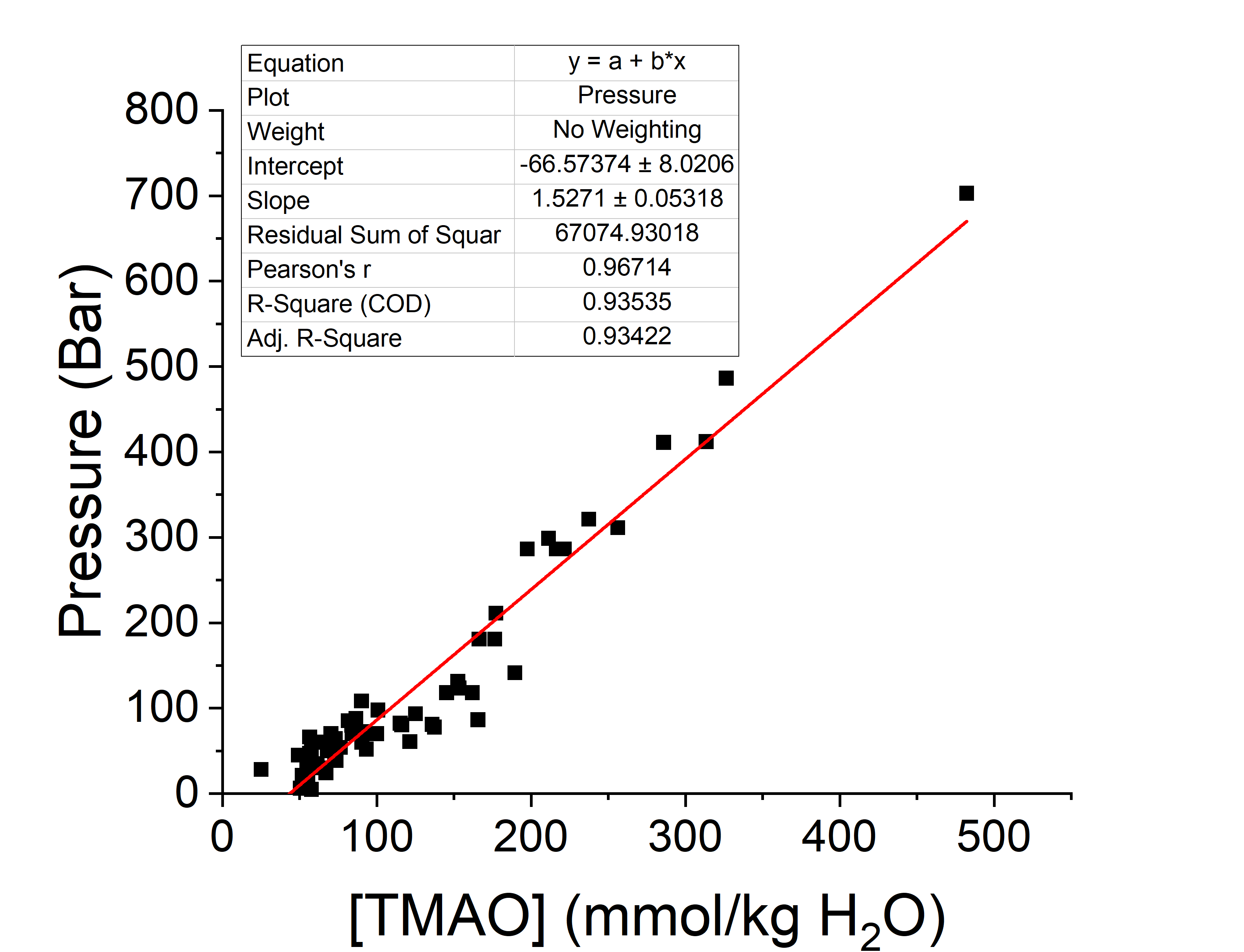


**Figure S13:** Replotting data for TMAO concentration in mmol/kg wet muscle tissue as a function of depth from which organism was harvested for teleosts as reported by Yancey *et al* ^3^, Bockus *et al* ^4^, and Samerotte *et al* ^5^ in figure 1 of main text as pressure from which the organism is harvested as a function of TMAO concentration in mmol/kg H_2_O.

**Note S6: Sample List**

To investigate pressure induced structural perturbations to water and aqueous TMAO a total of 10 samples were prepared. These are as follows, where H/D represents whether the chemical was fully protinated or fully deuterated, and HD represents an equimolar mixture of the two: H_2_O, D_2_O, HDO, H_2_O + H-TMAO, H_2_O + D-TMAO, H_2_O + HD-TMAO, D_2_O + H-TMAO, D_2_O + D-TMAO, D_2_O + HD-TMAO, HDO + HD-TMAO.

**Table S12:** Densities of pure water and aqueous TMAO at 2.0 mol/kg H_2_O at 25 bar and 4 kbar taken from previous literature^7–10^ and box dimensions of simulations.

| System | Density (g/cm^3^) | Density  (atoms/Å^3^) | Box dimension (Å) |
| --- | --- | --- | --- |
| H_2_O at 25 bar | 0.998 | 0.1000 | 53.1329 |
| H_2_O at 4 kbar | 1.131 | 0.1134 | 50.9518 |
| Aqueous TMAO at 25 bar | 1.002 | 0.0988 | 58.1729 |
| Aqueous TMAO at 4 kbar | 1.124 | 0.1109 | 57.9753 |


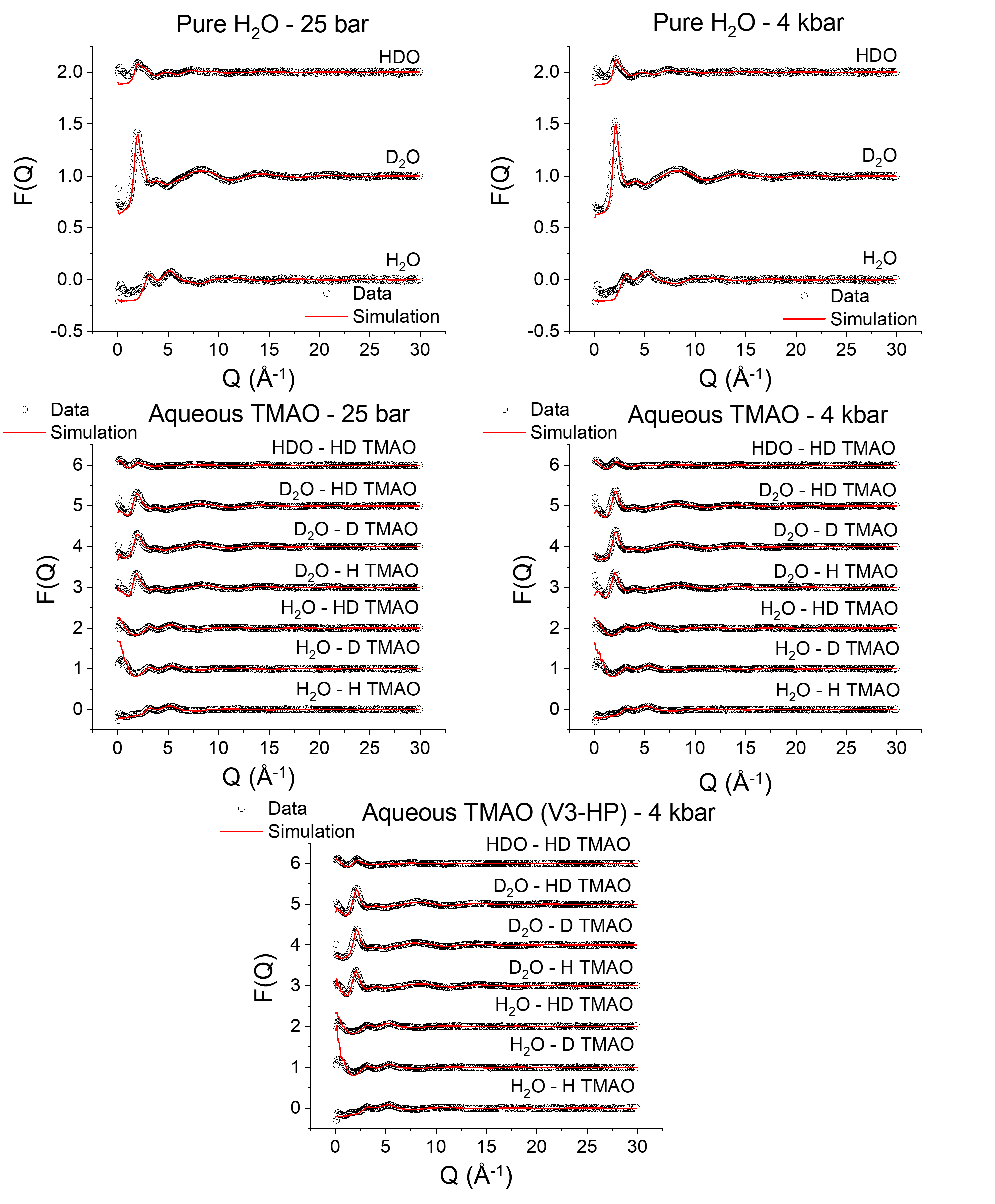


**Figure S14:** Supplied diffraction data *D*(*Q*) taken using NIMROD instrument (hollow circles) and total simulated structure factor *F*(*Q*) (red line) for three isotopic variants of pure water and seven isotopic variants of aqueous TMAO at 25 bar and 4 kbar. EPSR was performed using the potentials published by Meersman *et al*^1^ for all four samples and performed using the potential published by Hölzl^2^ *et al* for aqueous TMAO at 4 kbar. Each dataset vertically shifted by 1 for clarity.

**References**

(1) Meersman, F.; Bowron, D.; Soper, A. K.; Koch, M. H. J. Counteraction of Urea by Trimethylamine N-Oxide Is Due to Direct Interaction. *Biophys. J.* **2009**, *97* (9), 2559–2566. https://doi.org/10.1016/j.bpj.2009.08.017.

(2) Hölzl, C.; Kibies, P.; Imoto, S.; Frach, R.; Suladze, S.; Winter, R.; Marx, D.; Horinek, D.; Kast, S. M. Design Principles for High-Pressure Force Fields: Aqueous TMAO Solutions from Ambient to Kilobar Pressures. *J. Chem. Phys.* **2016**, *144* (14), 144104. https://doi.org/10.1063/1.4944991.

(3) Yancey, P. H.; Gerringer, M. E.; Drazen, J. C.; Rowden, A. A.; Jamieson, A. Marine Fish May Be Biochemically Constrained from Inhabiting the Deepest Ocean Depths. *Proc. Natl. Acad. Sci. U. S. A.* **2014**, *111* (12), 4461–4465. https://doi.org/10.1073/pnas.1322003111.

(4) Bockus, A. B.; Seibel, B. A. Trimethylamine Oxide Accumulation as a Function of Depth in Hawaiian Mid-Water Fishes. *Deep Sea Res. Part I Oceanogr. Res. Pap.* **2016**, *112*, 37–44. https://doi.org/10.1016/j.dsr.2016.03.005.

(5) Samerotte, A. L.; Drazen, J. C.; Brand, G. L.; Seibel, B. A.; Yancey, P. H. Correlation of Trimethylamine Oxide and Habitat Depth within and among Species of Teleost Fish: An Analysis of Causation. *Physiol. Biochem. Zool.* **2007**, *80* (2), 197–208. https://doi.org/10.1086/510566.

(6) LOVE, R. M. Water Content of Cod (Gadus Callarias L.) Muscle. *Nature* **1960**, *185* (4714), 692–692. https://doi.org/10.1038/185692a0.

(7) Engineering Toolbox. Water - Density, Specific Weight and Thermal Expansion Coefficient https://www.engineeringtoolbox.com/water-density-specific-weight-d_595.html (accessed Jul 8, 2021).

(8) Grindley, T.; Lind, J. E. PVT Properties of Water and Mercury. *J. Chem. Phys.* **1971**, *54* (9), 3983–3989. https://doi.org/10.1063/1.1675455.

(9) Makarov, D. M.; Egorov, G. I.; Kolker, A. M. Density and Volumetric Properties of Aqueous Solutions of Trimethylamine N-Oxide in the Temperature Range from (278.15 to 323.15) K and at Pressures up to 100 MPa. *J. Chem. Eng. Data* **2015**, *60* (5), 1291–1299. https://doi.org/10.1021/je500977g.

(10) Knierbein, M.; Held, C.; Hölzl, C.; Horinek, D.; Paulus, M.; Sadowski, G.; Sternemann, C.; Nase, J. Density Variations of TMAO Solutions in the Kilobar Range: Experiments, PC-SAFT Predictions, and Molecular Dynamics Simulations. *Biophys. Chem.* **2019**, *253* (May), 106222. https://doi.org/10.1016/j.bpc.2019.106222.
